# Integrated proteomic and transcriptomic profiles reveals the role of OAS3 in dermatomyositis pathogenesis

**DOI:** 10.3389/fimmu.2026.1735236

**Published:** 2026-01-26

**Authors:** Zhuangzhuang Zhang, Chenyu Zhuang, Hongjun Hao, Feng Gao, Zhaoxia Wang, Yun Yuan, Yiming Zheng

**Affiliations:** 1 Department of Neurology, Peking University First Hospital, Beijing, China; 2 Beijing Key Laboratory of Neurovascular Disease Discovery, Beijing, China; 3 Rare Diseases Medical Center, Peking University First Hospital, Beijing, China

**Keywords:** biomarker, dermatomyositis, OAS3, proteomics, transcriptomics, type I interferon

## Abstract

**Background:**

Dermatomyositis (DM) is an autoimmune myopathy often linked to aberrant type I interferon (IFN) pathway activation. However, the molecular mediators driving this IFN signature and their utility as biomarkers remain incompletely defined.

**Methods:**

We conducted an integrated multi-omics analysis combining plasma proteomics from 14 patients with DM and 5 healthy controls, with transcriptomic profiling of skeletal muscle derived from three publicly available Gene Expression Omnibus datasets (GSE11971, GSE1551, and GSE128470). Selected plasma proteins were further quantified and validated using enzyme-linked immunosorbent assay (ELISA).

**Results:**

Proteomic profiling identified 482 differentially expressed proteins (DEPs). Upregulated DEPs were enriched in antiviral responses and IFN-related immune pathways, while downregulated DEPs were associated with extracellular matrix organization. Transcriptomic analysis revealed 156 consistently upregulated differentially expressed genes across datasets, primarily involved in innate immunity, nucleic acid sensing, and antigen presentation. Integrative analysis identified 2′-5′-oligoadenylate synthetase 3 (OAS3) as a central hub within the IFN signaling network. ELISA validation demonstrated significantly elevated plasma OAS3 levels in DM patients (median: 5.073 ng/ml, IQR: 2.93–9.36) compared with controls (median: 2.723 ng/mL, IQR: 1.77–3.34), with a P-value of 0.018. Notably, plasma OAS3 levels showed a positive correlation with serum creatine kinase concentrations (r=0.55, P = 0.044).

**Conclusions:**

OAS3 expression was consistently elevated in both plasma and skeletal muscle tissues of individuals with DM. This molecule may act as a key enhancer of type I IFN-mediated pathogenic responses. Our results support the potential of OAS3 as a novel biomarker and therapeutic target in DM.

## Introduction

Dermatomyositis (DM) is an uncommon autoimmune myopathy primarily affecting skeletal muscle, characterized by progressive muscle weakness and unique cutaneous features (1, 2). In addition to skeletal muscle manifestations, DM is frequently associated with systemic complications, including interstitial lung disease (ILD), cardiac dysfunction, arthritis, and malignancy (3). The estimated incidence of DM varies between 1 and 15 cases per million individuals, with a reported prevalence ranging from 1.2 to 21 per 100,000 population (4). Patients with DM are estimated to have a nearly threefold increased mortality risk compared to the general population (5), with infection being the leading contributor to death, followed by ILD and malignancy (6). Despite improvements in diagnostic criteria, timely and accurate diagnosis of DM remains challenging, particularly in individuals lacking classical cutaneous lesions or evident muscle weakness (7). Early diagnosis is crucial, as the risk of malignancy is markedly elevated within the first three years of disease onset. As such, cancer screening has become an integral component of DM management and a major determinant of clinical prognosis (8, 9). Therefore, identifying molecular targets involved in DM pathogenesis is essential for facilitating early diagnosis and optimizing therapeutic strategies.

Advances in multi-omics technologies have enabled comprehensive investigations into the molecular landscape of DM, resulting in the identification of numerous candidate biomarkers and signaling pathways (10). Among these, dysregulated type I interferon (IFN) signaling has emerged as a central pathogenic mechanism in DM (11). Of particular interest is 2′-5′-oligoadenylate synthetase 3 (OAS3), an IFN-stimulated gene that recognizes double-stranded RNA and acts as a downstream effector of type I IFN signaling (12). Recent studies suggest that OAS3 may amplify IFN-mediated responses as a nucleic acid sensor, contributing to persistent immune activation and tissue damage in DM (13). Additionally, OAS3 has been implicated in other autoimmune conditions (14, 15). However, its specific role in the immunopathogenesis of DM remains poorly defined.

In this study, we conducted a comprehensive proteomic and transcriptomic analysis to identify key molecular drivers and signaling pathways involved in DM. Our findings highlight OAS3 as a potential biomarker and central mediator of IFN-driven pathology in DM, offering new insights into disease mechanisms and therapeutic targets.

## Methods

### Study design and participants

This research was designed as a single-center, retrospective study, enrolling 14 patients diagnosed with DM and 5 age- and sex-matched healthy individuals. All patients were diagnosed with DM according to the criteria established by the 119th European Neuromuscular Centre international workshop (16) and were recruited from Peking University First Hospital. According to the criteria, patients fulfilling the clinical, laboratory, and muscle biopsy criteria can be classified as possible DM even in the absence of typical cutaneous manifestations, a condition referred to as DM sine dermatitis. All patients with DM were newly diagnosed, had not received any immunosuppressive or corticosteroid treatment prior to blood sampling, and showed no evidence of malignancy upon comprehensive screening. Peripheral blood samples were collected from all subjects, and plasma was isolated for subsequent proteomic profiling and enzyme-linked immunosorbent assay (ELISA) validation. Clinical data, including demographic information, disease duration, clinical manifestations, and laboratory findings, were systematically documented. This study was approved by the Ethics Committee of Peking University First Hospital and conducted in accordance with the Declaration of Helsinki. Written informed consent was obtained from all participants prior to their inclusion in the study.

### Plasma proteomic analysis

Plasma samples were subjected to sequential protein extraction followed by enzymatic digestion. Samples were thawed at 4°C prior to protein extraction. Equal volumes of sample (100 μL) and beads (100 μL, 1 mg/mL) were mixed at 37°C for 1 hour with rotation. After binding, the beads–protein complexes were washed three times with 200 μL of wash buffer (10 mM Tris, pH 7.4; 150 mM KCl; 0.05% CHAPS). The washed beads were subsequently resuspended in lysis buffer containing 0.5% sodium deoxycholate (SDC, w/v), 10 mM tris(2-chloroethyl) phosphate (TCEP), 40 mM chloroacetamide (CAA), and 50 mM triethylammonium bicarbonate (TEAB). Protein extraction was achieved by sonication for 10 minutes, followed by centrifugation and transfer of the supernatant to a new tube. This extraction step was repeated once. The combined supernatants were then heated at 95°C for 10 minutes in a metal bath prior to downstream analysis. Peptides were subsequently analyzed using liquid chromatography coupled with tandem mass spectrometry. Separation was carried out on a Vanquish Neo UHPLC system (Thermo Fisher Scientific). The peptide mixture was initially dissolved in buffer A (0.1% formic acid) and loaded onto a reversed-phase trap column (Acclaim PepMap 100, Thermo Fisher Scientific), followed by chromatographic separation on a self-packed analytical column (17 cm length, 75 μm inner diameter). A linear gradient elution was applied using solvent A (0.1% formic acid in water) and solvent B (80% acetonitrile containing 0.1% formic acid), with the following proportions: 6.7–24% over 11.3 minutes, 24–36% for 5.7 minutes, 36–55% for 1.5 minutes, 55–99% for 0.5 minutes, and 99% for an additional 5 minutes. Mass spectrometric acquisition was performed using an Orbitrap Astral system (Thermo Fisher Scientific). Instrument settings included an ion source voltage of 2.2 kV, MS1 scanning from 380 to 980 m/z at a resolution of 240,000, and an MS2 isolation width of 2 m/z. MS2 scans covered the 150–2000 m/z range, with a scan time of 3 milliseconds and a normalized collision energy of 25. The automatic gain control target was set to 5e5.

The raw mass spectrometry data were converted to mzML format using MSConvert (v3.0.21072). Proteomic data analysis was performed using DIA-NN (version 1.8). Protein identification and quantification were conducted against the UniProt human reference proteome (uniprot_Homo_sapiens_UP000005640.fasta, 20,588 entries; downloaded on February 11, 2022) using the library-free quantification mode. Trypsin was specified as the proteolytic enzyme, allowing up to two missed cleavages, with a minimum peptide length of seven amino acids. Carbamidomethylation of cysteine was set as a fixed modification, while oxidation of methionine and N-terminal acetylation were included as variable modifications. The mass tolerance for both MS1 and MS2 was set to 20 ppm. False discovery rates (FDR) were controlled at 1%. The match between runs (MBR) option was enabled. After sequence database searching, Python (v3.10.4) was used for downstream preprocessing of the quantitative proteomics data, including normalization and missing value imputation. Protein intensities were normalized across samples using median-centering normalization. Subsequently, missing values were imputed using the row-wise half-minimum value method. Differentially expressed proteins (DEPs) were defined based on both statistical significance and magnitude of change. Proteins with a p value < 0.05 and a fold change ≥ 2 were considered upregulated, whereas proteins with a p value < 0.05 and a fold change ≤ 0.5 were considered downregulated. Given the relatively small sample size and the exploratory nature of this proteomic analysis, DEPs were identified based on nominal p values rather than FDR-adjusted p values, to retain potentially informative signals for downstream validation. Functional annotation of the DEPs was performed using the Gene Ontology (GO) and Kyoto Encyclopedia of Genes and Genomes (KEGG) databases. To identify central molecular pathways and hub proteins, enrichment analysis and construction of protein–protein interaction (PPI) networks were carried out.

### Transcriptomic analysis

Transcriptomic datasets of skeletal muscle tissue from DM patients, publicly available in the Gene Expression Omnibus database, were retrieved, including GSE11971, GSE1551, and GSE128470. The expression data were subjected to log2 transformation and normalization before downstream processing. To pinpoint differentially expressed genes (DEGs) between DM cases and healthy controls, differential analysis was carried out utilizing the limma package implemented in R (version 4.2.1). Genes meeting the criteria of |log2 fold change| ≥ 1 and adjusted p-value below 0.05 were deemed statistically significant. Commonly upregulated genes across the three datasets were determined using Venn diagram analysis.

Enrichment analysis of these shared DEGs was performed based on GO, KEGG, and Reactome pathway annotations using R. Only enrichment results with adjusted p-values less than 0.05 were retained for interpretation. PPI networks were generated through the STRING database (version 11.5), with a minimum required interaction confidence score set at 0.4. Final network visualization and further exploration were performed using Cytoscape (v3.9.1).

### ELISA validation

To validate the multi-omics findings, plasma protein concentrations were quantified in the same group of 14 DM patients using a commercially available human ELISA kit, following the protocol provided by the manufacturer. Each plasma sample was assessed in duplicates. Optical density readings were taken at 450 nm with a microplate reader, and the protein concentrations were calculated using a standard calibration curve.

### Statistical analysis

All data analyses were performed using R software (version 4.2.1) and GraphPad Prism (version 10.2.0). Categorical variables were presented as counts and percentages, and compared using the chi-square test. Continuous variables were expressed as medians with interquartile ranges and analyzed using the Mann–Whitney U test. Volcano plots and enrichment analyses were visualized using R, and PPI networks were visualized through the STRING platform (v11.5) and Cytoscape (v3.9.1). A two-tailed p-value < 0.05 was considered indicative of statistical significance.

To account for the overall activation of type I IFN signaling, an interferon composite score was calculated for each sample based on representative interferon-stimulated proteins. Specifically, the score was defined as the geometric mean of median-centered normalized plasma abundances of GBP1, EPSTI1, IFI6, CXCL10, IFI27, IFIT5, IFIT1, DDX60, ISG15, SIGLEC1, OAS2, and MX1, which collectively capture core type I IFN activity. This composite score was subsequently used in adjusted analyses to evaluate the association between OAS3 and serum creatine kinase (CK) independent of global interferon activation.

## Results

### Demographic and clinical features of study participants

Fourteen individuals diagnosed with DM were enrolled in the analysis. Their baseline features are summarized in **Table 1**. Among them, 5 patients (35.7%) were male. The median age at onset was 55.5 years (IQR: 33.75–67.5), and the median disease duration was 7 months (IQR: 3–12.25). The most frequently observed clinical symptoms were limb weakness (85.7%), myalgia (78.6%), dysphagia (35.7%), and dyspnea (7.1%). With respect to extramuscular involvement, fever was observed in 6 patients (42.9%). Cutaneous manifestations were common, with 12 patients (85.7%) exhibiting skin rash, including Gottron’s sign in 10 patients (71.4%), heliotrope rash in 9 patients (64.3%), mechanic’s hands in 2 patients (14.3%), and cutaneous ulcerations in 4 patients (28.6%). In addition, gastroesophageal reflux was present in 1 patient (7.1%). Systemic involvement included cardiac involvement in 5 patients (35.7%) and renal involvement in 4 patients (28.6%). Arthralgia was reported in 3 patients (21.4%), and ILD was diagnosed in 5 patients (35.7%). Laboratory testing revealed a median serum CK level of 1130 IU/L (IQR: 799.5–2456.3). Antinuclear antibodies were positive in 7 patients (50.0%). Regarding myositis-specific autoantibodies, 4 patients tested positive for anti-NXP2 antibody, 3 for anti-Mi-2 antibody, 4 for anti-TIF1-γ antibody, and 3 for anti-MDA5 antibody. Two patients did not present with typical skin rash but met the criteria for DM sine dermatitis according to the ENMC classification. The control group consisted of 5 healthy individuals, including 4 males. The median age in the control group was 59.0 years (IQR: 58.0–62.0). No significant differences in age or gender composition were found between DM and control group (P > 0.05).

**Table 1:** Clinical characteristics of patients with DM.

Characteristics	DM(n=14)
Male, n (%)	5 (35.71%)
Age at disease onset, median (IQR), years	55.5 (33.75, 67.5)
Disease duration, median (IQR), months	7 (3-12.25)
Muscle weakness	
Limb weakness, n (%)	12 (85.71%)
Dyspnea, n (%)	1 (7.1%)
Dysphagia, n (%)	5 (35.71%)
Myalgia, n (%)	11 (78.6%)
Extramuscular symptoms	
Fever, n (%)	6 (42.86%)
Skin rash, n (%)	12 (85.7%)
Gottron’s sign, n (%)	10 (71.43%)
Heliotrope rash, n (%)	9 (64.29%)
Mechanic’s hand, n (%)	2 (14.29%)
Cutaneous ulcerations, n (%)	4 (28.57%)
Gastroesophageal reflux, n (%)	1 (7.14%)
Cardiac involvement, n (%)	5 (35.71%)
Renal involvement, n (%)	4 (28.57%)
Arthralgia, n (%)	3 (21.4%)
Interstitial lung disease, n (%)	5 (35.71%)
Blood examination	
Serum CK, median (IQR), IU/L	1130 (799.5, 2456.25)
Positive ANA, n (%)	7 (50%)
Myositis specific autoantibody	
anti-NXP2 antibody	4 (28.6%)
anti-Mi-2 antibody	3 (21.4%)
anti-TIF1-γ antibody	4 (28.6%)
anti-MDA5 antibody	3 (21.4%)

ANA, antinuclear antibody; CK, creatine kinase; DM, dermatomyositis; IQR, interquartile range; MDA5, melanoma differentiation-associated protein 5; NXP2, nuclear matrix protein 2; TIF1-γ, transcription intermediary factor 1-gamma.

### Plasma proteomic analysis

**Figure 1:**
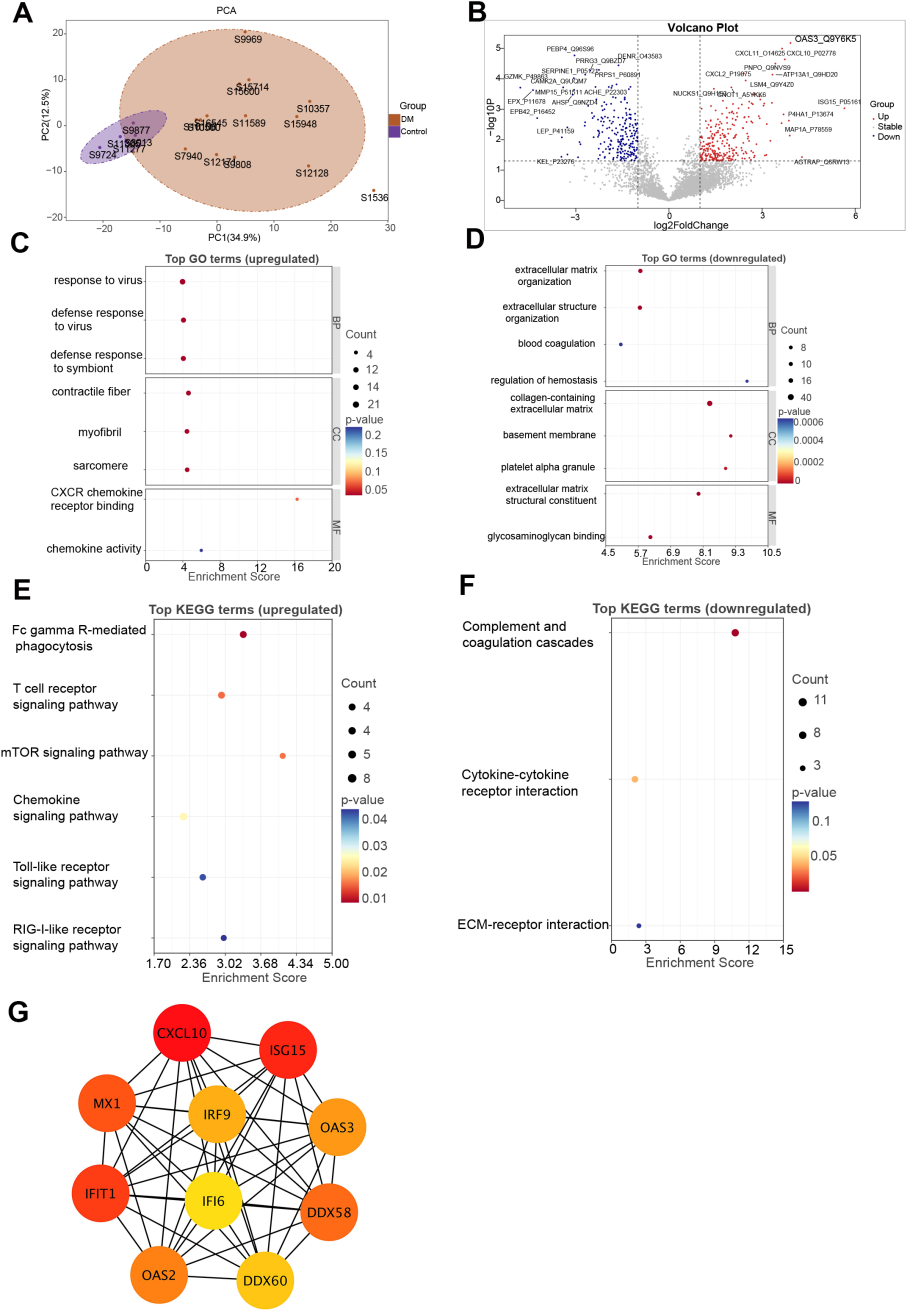
Proteomic analysis of DM. **(A)** PCA of proteomic profiles showing clear separation between DM patients and healthy controls. **(B)** Volcano plot of DEPs. Red and blue dots represent significantly upregulated and downregulated proteins in DM, respectively. **(C, D)** GO enrichment analysis of upregulated **(C)** and downregulated **(D)** proteins. **(E, F)** KEGG pathway enrichment analysis of upregulated **(E)** and downregulated **(F)** proteins. **(G)** PPI network constructed from DEPs. DEPs, differentially expressed proteins; DM, dermatomyositis; GO, Gene Ontology; KEGG, Kyoto Encyclopedia of Genes and Genomes; PCA, principal component analysis; PPI, protein–protein interaction.

Proteomic profiling was conducted on plasma obtained from 14 patients with DM and 5 healthy controls. Principal component analysis revealed clear separation between the DM and control groups, indicating good data quality and experimental reproducibility (**Figure 1A**). In total, 482 DEPs were detected, with 260 showing increased expression and 222 reduced expression levels (**Figure 1B**). A complete list of DEPs is provided in **Supplementary Table S1**. GO functional enrichment indicated that upregulated DEPs were significantly associated with antiviral responses and cytoskeletal regulation, including response to virus, defense response to virus, defense response to symbiont, contractile fiber, myofibril, sarcomere, CXCR chemokine receptor binding, and chemokine activity (**Figure 1C**). In contrast, downregulated DEPs were enriched in GO terms such as extracellular matrix organization, extracellular structure organization, blood coagulation, regulation of hemostasis, collagen-containing extracellular matrix, basement membrane, platelet alpha granule, extracellular matrix structural constituent, and glycosaminoglycan binding (**Figure 1D**). Furthermore, KEGG-based pathway analysis highlighted that several infection- and immune-related pathways were significantly upregulated in DM, including Fc gamma R-mediated phagocytosis, T cell receptor signaling pathway, mTOR signaling pathway, chemokine signaling pathway, Toll-like receptor signaling pathway, and RIG-I-like receptor signaling pathway (**Figure 1E**). In contrast, pathways such as complement and coagulation cascades, cytokine-cytokine receptor interaction, and ECM-receptor interaction were significantly downregulated (**Figure 1F**). The complete GO and KEGG enrichment results are provided in **Supplementary Figure S1**.

Through PPI network construction, the 10 most interconnected hub proteins among the DEPs were identified, suggesting their potential central roles in DM pathogenesis. Hub proteins were selected utilizing the degree centrality metric via the cytoHubba plugin integrated in Cytoscape. Notably, most hub proteins—including IRF9, ISG15, MX1, IFIT1, OAS2, OAS3, and DDX58—are involved in the type I IFN signaling pathway, underscoring its critical role in the immunopathogenesis of DM (**Figure 1G**).

Among the DEPs, ISG15 (log_2_FC = 5.65), AGTRAP (log_2_FC = 4.28), and OAS3 (log_2_FC = 3.91) were the three most upregulated proteins (**Figure 1B**). Notably, OAS3 demonstrated a substantial increase in abundance together with consistent statistical support. To further contextualize the relevance of OAS3 within the IFN-stimulated proteins, we performed a direct comparison with representative IFN-stimulated proteins detected in the plasma proteomic dataset, including ISG15, MX1, IFIT1, and CXCL10. As summarized in **Supplementary Table S2** and illustrated in **Supplementary Figure S2**, OAS3 showed robust upregulation comparable to or exceeding that of representative IFN-stimulated proteins. Although multiple IFN-stimulated proteins were upregulated in DM, OAS3 was prioritized for further investigation based on several considerations. First, OAS3 is a core effector of the 2′–5′-oligoadenylate synthetase–Ribonuclease L (RNase L) pathway, which represents a downstream antiviral effector arm of type I IFN signaling rather than a proximal transcriptional response. Second, OAS3 was consistently upregulated and identified as a hub molecule in both plasma proteomic and subsequent skeletal muscle transcriptomic analyses, indicating cross-tissue and cross-omics concordance. Third, OAS3 exhibited extensive connectivity within the PPI network, suggesting a central regulatory role within the IFN-driven immune landscape.

### Transcriptomic analysis

**Figure 2:**
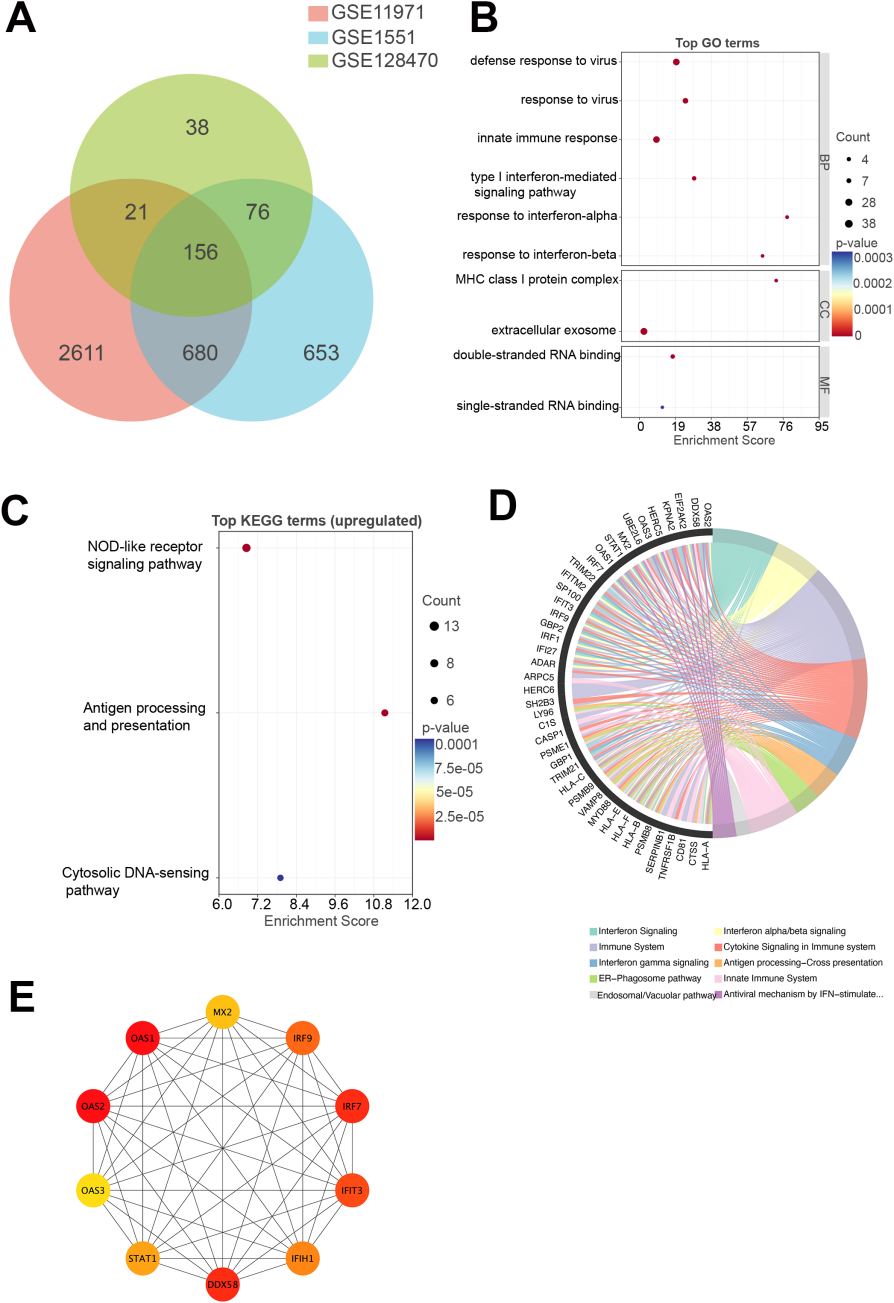
Transcriptomic analysis of DM. **(A)** Venn diagram illustrating the number of commonly upregulated genes across three GEO transcriptomic datasets (GSE11971, GSE1551, and GSE128470) derived from skeletal muscle tissue of DM patients. A total of 156 genes were consistently upregulated across all datasets. **(B)** GO enrichment analysis of the commonly upregulated genes. **(C)** KEGG pathway enrichment analysis of the commonly upregulated genes. **(D)** Reactome pathway enrichment analysis of the commonly upregulated genes. **(E)** PPI network constructed from the commonly upregulated genes. DM, dermatomyositis; GEO, Gene Expression Omnibus; GO, Gene Ontology; KEGG, Kyoto Encyclopedia of Genes and Genomes; PPI, protein–protein interaction.

To identify shared transcriptional alterations in DM, a Venn diagram was constructed to determine commonly upregulated DEGs across three independent skeletal muscle transcriptomic datasets (GSE11971, GSE1551, and GSE128470) (**Figure 2A**). A total of 156 genes were consistently upregulated across all three datasets. Specifically, GSE11971, GSE1551, and GSE128470 contained 3,468, 1,565, and 291 upregulated genes, respectively.

Among the 156 shared upregulated genes, several components of the OAS3–RNase L–type I interferon (IFN-I) signaling axis were prominently represented. These included members of the OAS family (OAS1, OAS2, OAS3), RNA sensors in the RIG-I-like receptor pathway (DDX58, IFIH1, DDX60, DDX21), and downstream transcriptional regulators (IRF1, IRF7, IRF9, STAT1). In addition, numerous IFN-stimulated genes, such as IFI27, IFI35, IFI44, IFI44L, IFIT3, IFIT5, IFITM1, IFITM2, IFITM3, MX2, USP18, ZC3HAV1, and SAMHD1, were also consistently upregulated, along with regulatory molecules including ADAR and EIF2AK2. Although RNASEL itself was not detected, the coordinated upregulation of both upstream and downstream effectors strongly supports the activation of the OAS3–RNase L–IFN-I positive feedback loop in DM muscle tissue.

GO enrichment analysis revealed that the upregulated DEGs were significantly associated with biological processes and molecular functions related to antiviral immunity and IFN signaling, including defense response to virus, response to virus, innate immune response, type I interferon-mediated signaling pathway, response to interferon-alpha, response to interferon-beta, MHC class I protein complex, extracellular exosome, double-stranded RNA binding, and single-stranded RNA binding (**Figure 2B**). These results suggest a dominant role for innate immune activation in DM muscle tissue. KEGG pathway analysis further confirmed that the 156 upregulated genes were significantly enriched in immune- and infection-related pathways, including NOD-like receptor signaling pathway, antigen processing and presentation, cytosolic DNA-sensing pathway (**Figure 2C**). Reactome pathway enrichment analysis highlighted key pathways associated with IFN signaling, cytokine signaling in the immune system, and antigen processing–cross presentation (**Figure 2D**), collectively reinforcing the presence of a strong type I IFN-driven transcriptional program in DM. The complete GO and KEGG enrichment results are provided in **Supplementary Figure S3**.

To identify core regulatory nodes, a PPI network was established using STRING, and hub genes were ranked via the cytoHubba plugin within Cytoscape, leveraging degree centrality metrics. The top 10 hub genes included OAS1, OAS2, OAS3, IRF7, IRF9, DDX58, IFIH1, IFIT3, MX2, and STAT1 (**Figure 2E**). These genes are primarily involved in IFN-mediated immune signaling and the recognition of viral nucleic acids, further supporting the concept of a type I IFN signature as a central molecular feature of DM.

### Validation of OAS3 expression and clinical correlation

**Figure 3:**
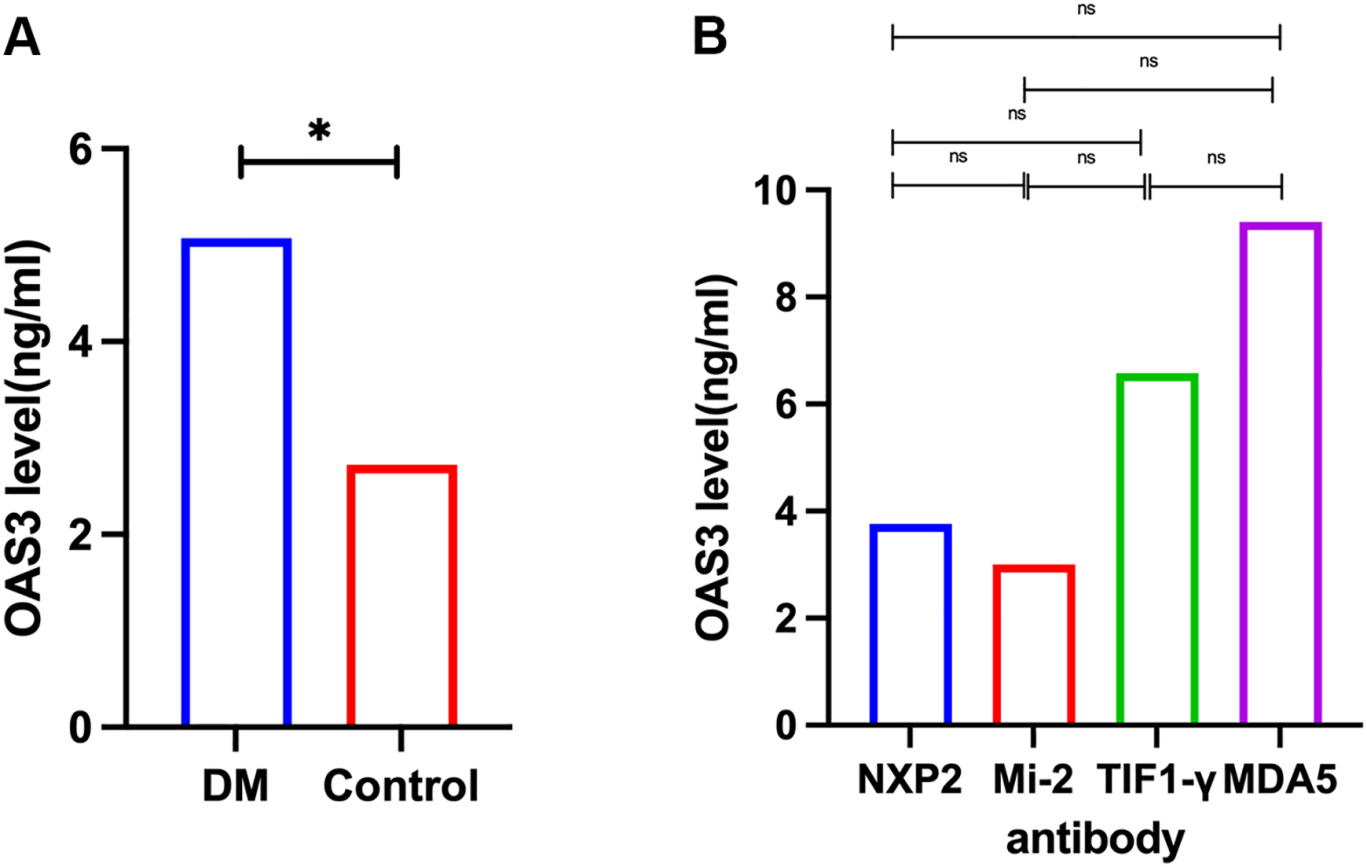
Quantification of plasma OAS3 levels by ELISA. **(A)** Plasma OAS3 concentrations were significantly elevated in patients with DM compared to healthy controls. **(B)** Plasma OAS3 concentrations stratified by different myositis-specific autoantibodies (anti-NXP2 antibody, anti-Mi-2 antibody, anti-TIF1-γ antibody, anti-MDA5 antibody) showed no significant differences among subgroups. DM, Dermatomyositis; ELISA, Enzyme-Linked Immunosorbent Assay; MDA5, melanoma differentiation-associated protein 5; NXP2, nuclear matrix protein 2; OAS3, 2’-5’-Oligoadenylate Synthetase 3; TIF1-γ, transcription intermediary factor 1-gamma. The asterisk (*) indicates a statistically significant difference with P < 0.05.

**Figure 4:**
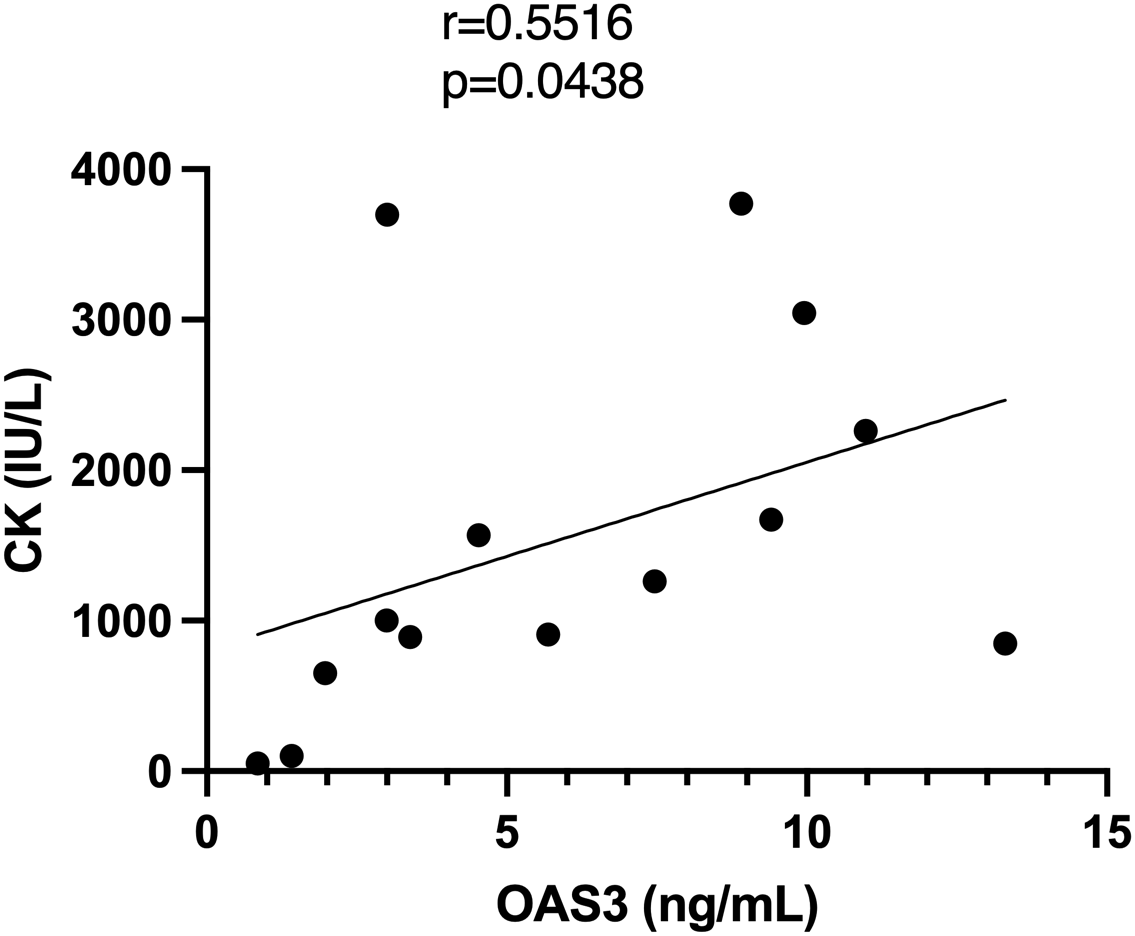
Correlation analysis between plasma OAS3 levels and serum CK. CK, creatine kinase; OAS3, 2’-5’-Oligoadenylate Synthetase 3.

Plasma proteomic analysis identified OAS3 as one of the most significantly upregulated proteins in DM and as a central hub in the PPI network. Consistent findings were observed in the skeletal muscle transcriptomic analysis, where OAS3 was markedly upregulated and ranked among the top hub genes. Based on these integrated multi-omics findings, OAS3 was selected for further validation and clinical correlation analysis. Plasma OAS3 concentrations were quantified using ELISA. The results indicated a significantly higher concentration of OAS3 in the DM group (median: 5.073 ng/mL, IQR: 2.93–9.36) relative to healthy individuals (median: 2.723 ng/mL, IQR: 1.77–3.34), with a P-value of 0.018 (**Figure 3A**). Subgroup analysis revealed no statistically significant differences in plasma OAS3 concentrations among DM patients stratified by different myositis-specific autoantibodies (**Figure 3B**). Additionally, plasma OAS3 concentration showed a positive correlation with serum CK levels (r = 0.5516, P = 0.0438), suggesting a potential association between OAS3 expression and disease activity (**Figure 4**). The individual plasma OAS3 and CK values for each patient are provided in **Supplementary Table S3**. After adjustment for the interferon composite score, the association between plasma OAS3 levels and serum CK was attenuated but remained directionally consistent, as demonstrated by partial correlation analysis using residuals (**Supplementary Figure S4**). Consistent results were obtained in a multivariable linear regression model incorporating both OAS3 and the interferon composite score (**Supplementary Table S4**).

## Discussion

In this study, we integrated plasma proteomics, transcriptomic profiling of skeletal muscle from publicly available datasets, and ELISA-based validation to demonstrate that OAS3 is consistently and significantly upregulated in DM. Our findings reveal a strong association between OAS3 expression and activation of the type I IFN signaling pathway, implicating OAS3 as a potential key regulatory molecule in the immunopathogenesis of DM.

Our proteomic analysis identified 482 DEPs, with functional enrichment revealing significant involvement in pathways related to antiviral immunity and extracellular matrix organization. Notably, a substantial proportion of upregulated DEPs, including OAS2, OAS3, IRF9, DDX58, and MX1, were enriched in the type I IFN signaling pathway. This observation is consistent with previous multi-omics studies in DM, which have repeatedly demonstrated robust activation of type I IFN signaling, a process thought to contribute to immune-mediated muscle injury and characteristic skin manifestations (10, 17–19). In contrast, the downregulation of extracellular matrix associated proteins, such as collagens and basement membrane components, likely reflects tissue remodeling and structural degradation secondary to chronic inflammation (20, 21). Transcriptomic analysis of skeletal muscle from three independent Gene Expression Omnibus datasets further confirmed the upregulation of IFN-related pathways in DM. We identified 156 commonly upregulated genes, many of which are involved in innate antiviral responses, nucleic acid sensing, and antigen presentation. The convergence of proteomic and transcriptomic findings strongly supports the central role of type I IFN signaling in DM pathogenesis. Among the identified hub genes, OAS3 emerged as a particularly notable candidate, showing significant upregulation in the plasma proteomic dataset and consistent overexpression across both plasma proteomics and muscle transcriptomics. In addition, OAS3 exhibited extensive interactions with other IFN-related proteins in the PPI network. Functionally, OAS3 is a type I IFN–inducible nucleic acid sensor that recognizes endogenous RNA, activates RNase L, and promotes the release of immunogenic RNA fragments, thereby amplifying IFN signaling (22, 23). Beyond its canonical antiviral role, recent evidence suggests that sustained OAS3 activation may contribute to excessive immune activation and tissue damage in autoimmune diseases such as systemic lupus erythematosus and psoriasis (14, 15). Our findings extend this paradigm to DM, suggesting that OAS3 may serve as a key amplifier of pathogenic IFN signaling. ELISA-based validation confirmed significantly elevated plasma OAS3 levels in DM patients compared with healthy controls, supporting its potential utility as a circulating biomarker.

**Figure 5:**
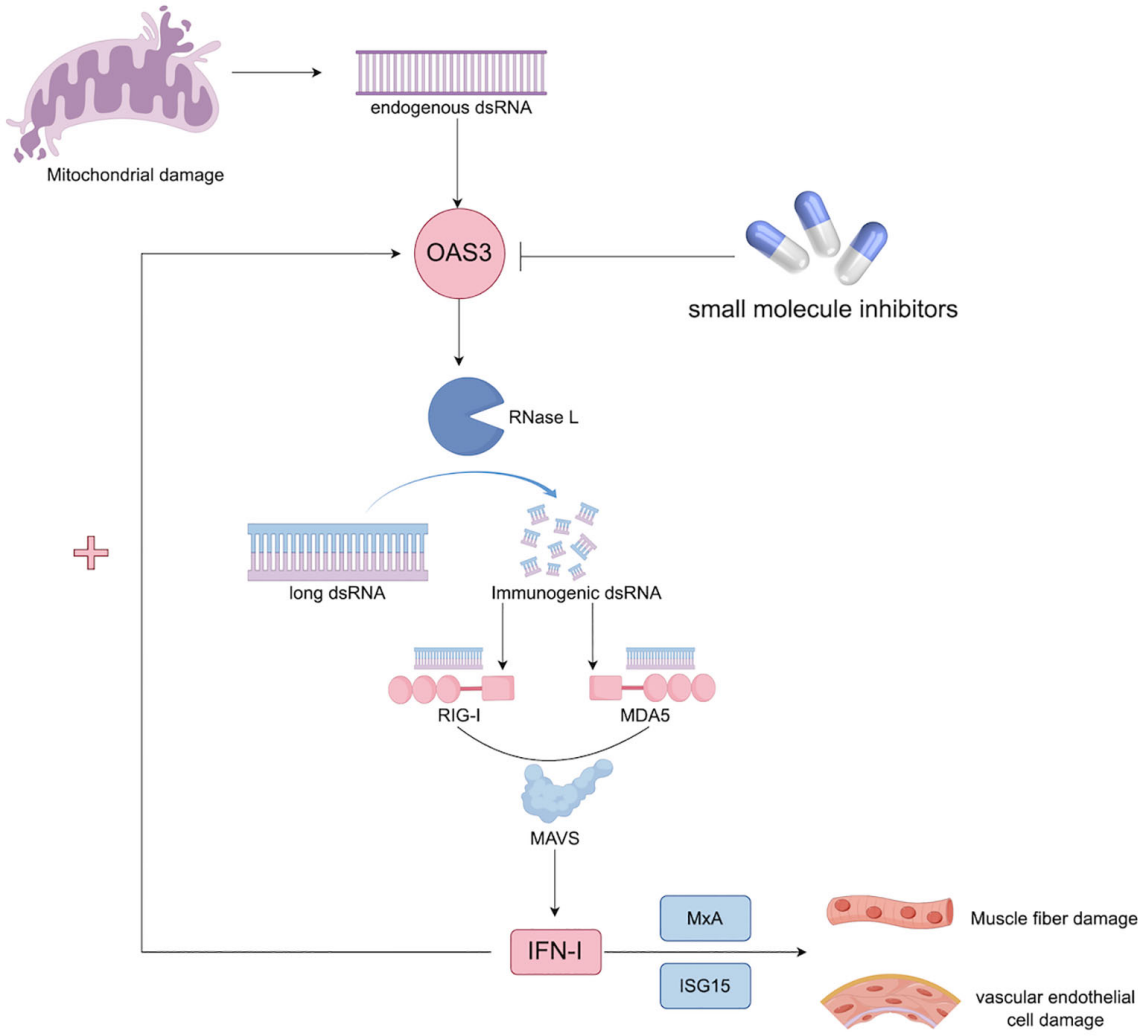
Hypothesis of the OAS3–RNase L–IFN-I axis in the pathogenesis of DM. Excessive activation of the OAS–RNase L system can induce collateral degradation of host RNA, thereby impairing muscle cell viability and regenerative capacity (24, 25). In parallel, OAS3 may amplify type I IFN signaling, promoting sustained inflammatory responses in muscle and skin tissues (26). Furthermore, RNase L activation generates self-derived RNA fragments that stimulate cytosolic RNA sensors, including DDX58 and IFIH1, establishing a feed-forward loop that perpetuates type I IFN signaling (27). dsRNA, double-stranded RNA; IFN, interferon; ISG15, Interferon-stimulated gene 15; MAVS, mitochondrial antiviral signaling; MDA5, melanoma differentiation-associated protein 5; MxA, myxovirus resistance protein A; OAS3, 2’-5’-Oligoadenylate Synthetase 3; RIG-I, Retinoic acid-inducible gene I; RNase L, Ribonuclease L.

Mechanistically, the upregulation of OAS3 may contribute to the pathogenesis of DM through multiple interrelated pathways. First, excessive activation of the OAS–RNase L system may lead to collateral degradation of host RNA, impairing muscle cell viability and regenerative capacity (24, 25). Second, OAS3-mediated amplification of IFN-I signaling may promote persistent inflammation in both muscle and skin tissues (26). Third, activation of RNase L by OAS3 generates small self-RNA fragments that further stimulate cytosolic RNA sensors such as DDX58 and IFIH1, thereby establishing a self-perpetuating feed-forward loop that sustains IFN-I signaling (27). The proposed pathogenic role of the OAS3–RNase L–IFN-I axis in DM is illustrated in **Figure 5**. Importantly, therapeutic strategies targeting the IFN pathway are already under investigation in DM, with JAK inhibitors demonstrating promising efficacy in dampening IFN-driven inflammation by blocking downstream JAK–STAT signaling (28, 29). Within this therapeutic framework, OAS3 represents a potential upstream regulator of IFN amplification. Targeting OAS3 directly, or modulating RNase L activity, may offer a complementary approach to current therapies. By intervening at an earlier stage of the IFN cascade, OAS3-targeted therapy may more selectively attenuate pathological IFN responses, potentially mitigating chronic inflammation and tissue injury while preserving broader host immune function. Although the association between plasma OAS3 levels and serum CK was attenuated after adjustment for an interferon composite score, this observation is biologically plausible and consistent with the role of OAS3 as a downstream effector within the IFN signaling network, in which IFN-stimulated proteins are intrinsically co-regulated.

This study has several limitations. First, the proteomic analysis was conducted in a relatively small cohort, primarily due to the low prevalence of DM, which inherently limits patient recruitment. Second, the plasma proteomic analysis was designed as an exploratory screening approach and therefore relied on nominal p values rather than adjusted p values, given the limited sample size. Importantly, the principal findings were independently supported by skeletal muscle transcriptomic datasets and ELISA-based validation, which strengthens the overall robustness of the conclusions. Third, functional validation of OAS3 in the context of DM pathogenesis was not performed *in vitro* or *in vivo*. Therefore, although our multi-omics analyses reveal strong associations, additional mechanistic studies are necessary to establish causality and to further evaluate the therapeutic potential of targeting OAS3 in DM. Finally, the relatively small sample size may have limited the statistical power of multivariable analyses. The attenuated association between plasma OAS3 levels and serum CK after adjustment for the interferon composite score may reflect shared biological variance among interferon-stimulated proteins, which are intrinsically co-regulated within the type I IFN signaling network.

Future research should aim to validate OAS3 as a biomarker in larger, multicenter cohorts to enhance the generalizability of findings. Mechanistic investigations using cell-based and animal models are needed to elucidate how OAS3 overexpression contributes to muscle and skin pathology, and to evaluate whether targeting OAS3 or its downstream signaling components can mitigate disease progression. In addition, incorporating other omics modalities, such as metabolomic profiling and single-cell RNA sequencing, could offer deeper insights into the IFN-mediated pathogenic mechanisms in DM and help identify new therapeutic targets.

## Conclusions

In summary, our integrative proteomic and transcriptomic analyses identify OAS3 as a pivotal component of the type I IFN–mediated immune response in DM. Its consistent upregulation in both plasma and skeletal muscle highlights its potential as a biomarker and therapeutic target. These results not only deepen our insight into the mechanisms underlying DM but also lay the groundwork for designing precision diagnostic approaches and personalized treatment interventions.

## Data Availability

The raw data supporting the conclusions of this article will be made available by the authors, without undue reservation.
