# Supplementary material for: Integrated proteomic and transcriptomic profiles reveals the role of OAS3 in dermatomyositis pathogenesis
Source: Front Immunol. 2026 Jan 26;17:1735236. doi: 10.3389/fimmu.2026.1735236 (PMC12883356; doi:10.3389/fimmu.2026.1735236)
Supplement: Supplementary file 1 [file Table1.docx]

**Supplementary material**

**Table S1. Full list of differentially expressed proteins identified in plasma proteomic analysis.**

| Gene symbol | log₂ fold change | Nominal P value | adjusted P value |
| --- | --- | --- | --- |
| COQ9 | 1.00067118 | 0.03225749 | 0.30264907 |
| DNASE1L3 | -1.1911222 | 0.03028499 | 0.29497307 |
| RANGAP1 | -1.0955289 | 0.00632129 | 0.14530851 |
| OAS3 | 3.9145691 | 6.64E-06 | 0.02204773 |
| GTDC1 | 1.20486768 | 0.01953192 | 0.24593232 |
| COL18A1 | -1.3180448 | 0.02429567 | 0.27271414 |
| ISG15 | 5.65402931 | 0.00093112 | 0.06794423 |
| ETHE1 | 2.18814621 | 0.00131995 | 0.0799415 |
| PRAP1 | -1.5592163 | 0.01300337 | 0.2053062 |
| PCOLCE | -1.0463344 | 0.03860091 | 0.32279336 |
| WASHC3 | 1.07658828 | 0.04252605 | 0.33895993 |
| HRC | 2.37967993 | 0.00412623 | 0.11556518 |
| PFKM | -1.2243837 | 0.0134226 | 0.2074693 |
| ARHGAP25 | 2.05551204 | 0.02280533 | 0.26347436 |
| MCTS1 | -1.1393227 | 0.00394828 | 0.11434218 |
| IGFBP6 | -2.0153923 | 0.0006981 | 0.06085176 |
| CMTM3 | 1.30495235 | 0.00881896 | 0.16594387 |
| SPINK5 | -1.9224742 | 0.00399152 | 0.11482903 |
| GKN2 | -2.3398004 | 0.00047462 | 0.05651512 |
| MIGA2 | 1.0679386 | 0.03153039 | 0.30102859 |
| NIPSNAP2 | 1.73861787 | 0.00052568 | 0.06009398 |
| PRRG3 | -2.5366772 | 5.34E-05 | 0.02889205 |
| FLOT1 | -2.4277195 | 0.00830677 | 0.16391523 |
| NOTUM | -1.4289191 | 0.0485735 | 0.35777959 |
| SEC62 | 2.16794245 | 0.01787309 | 0.23445073 |
| RFT1 | 1.35118692 | 0.00390077 | 0.11434218 |
| L3HYPDH | 1.26074881 | 0.01917495 | 0.24426974 |
| ATL1 | 1.93773047 | 0.01592847 | 0.22177333 |
| PCSK1N | -1.9375081 | 0.00887686 | 0.16594387 |
| OGA | 1.61067538 | 0.02062085 | 0.24951803 |
| MSRA | 1.81982592 | 0.00807113 | 0.16342891 |
| CBL | 2.40063949 | 0.02871406 | 0.28558708 |
| RTF1 | 2.58780994 | 0.00056357 | 0.06085176 |
| SPATA17 | -1.9098986 | 0.00238592 | 0.09253963 |
| PON1 | -1.2703354 | 0.00147249 | 0.08326867 |
| FBLN7 | -1.5531299 | 0.00759593 | 0.15868707 |
| SCUBE1 | -1.5482105 | 0.02783179 | 0.28558708 |
| CAMK2A | -3.066308 | 9.53E-05 | 0.03448464 |
| KTN1 | 2.35295519 | 0.02263526 | 0.26289705 |
| BCLAF1 | 1.50423355 | 0.00109474 | 0.07205381 |
| DEK | 1.34635492 | 0.00229563 | 0.0914884 |
| CFD | -1.774482 | 0.00178075 | 0.08761099 |
| CEP131 | 2.42542667 | 0.00186771 | 0.08761099 |
| GART | 2.0830165 | 0.02209878 | 0.25875229 |
| TMOD1 | -2.2644313 | 0.019782 | 0.24622634 |
| ALDH18A1 | 2.13238334 | 0.00751256 | 0.15868707 |
| UBXN4 | 1.34183489 | 0.00535303 | 0.1328775 |
| SLC27A3 | 1.94651882 | 0.00130482 | 0.0799415 |
| RAN | -1.0706304 | 0.00546954 | 0.13499826 |
| CLU | -1.0620495 | 0.00327168 | 0.10224589 |
| LFNG | -2.1879773 | 0.00250062 | 0.0937479 |
| CHID1 | -1.1863177 | 0.00749264 | 0.15868707 |
| F7 | -1.1138291 | 0.00721744 | 0.15676278 |
| THEM6 | 1.07851186 | 0.01648315 | 0.22447308 |
| RPS23 | -1.2387512 | 0.02565432 | 0.28071127 |
| PIK3R4 | 1.69418784 | 0.0029511 | 0.1001528 |
| RAB12 | 1.57663337 | 0.00213644 | 0.08923727 |
| CHAD | -1.526452 | 0.02103943 | 0.25247322 |
| BAIAP3 | -3.2726045 | 0.0307355 | 0.29662074 |
| SLC33A1 | 1.36183649 | 0.00382073 | 0.11290637 |
| PIK3CG | 1.46688651 | 0.00661817 | 0.14896022 |
| DNAJC2 | -1.2722775 | 0.01868803 | 0.24017995 |
| GPR15L | -2.078871 | 0.00100068 | 0.07011198 |
| LAP3 | 2.39070646 | 0.0041917 | 0.11592883 |
| IGFBP4 | -1.197724 | 0.00621335 | 0.14530851 |
| SWAP70 | 1.04789497 | 0.04085766 | 0.33485469 |
| TTC9C | 1.65885861 | 0.00885852 | 0.16594387 |
| COMMD5 | 1.35628995 | 0.00078559 | 0.06319625 |
| CRYAB | 2.54013405 | 0.00922414 | 0.16727683 |
| ARMT1 | 1.51753267 | 0.02911484 | 0.28837154 |
| LRRC59 | 1.47090564 | 0.03072984 | 0.29662074 |
| FETUB | -1.1368265 | 0.0091038 | 0.16616352 |
| DUSP23 | 1.68518383 | 0.03292659 | 0.30439955 |
| CSNK1A1 | -1.6969847 | 0.00683869 | 0.15273275 |
| FGL1 | 1.52670155 | 0.04637669 | 0.35546504 |
| USE1 | 2.01711633 | 0.00127181 | 0.0799415 |
| MKKS | 2.24172915 | 0.00093038 | 0.06794423 |
| CTSH | -1.1656365 | 0.01157932 | 0.19110527 |
| SLPI | -1.2668453 | 0.00366172 | 0.1097001 |
| ADH1B | -2.1272181 | 0.0253996 | 0.2793313 |
| RNASE4 | -1.6022369 | 0.01187286 | 0.19244668 |
| INPP5B | 1.43500732 | 0.04628215 | 0.35546504 |
| NUCKS1 | 1.44719339 | 0.00021275 | 0.03931168 |
| BMP1 | -1.2386948 | 0.00191306 | 0.08761099 |
| MT-ND4 | 1.76823647 | 0.02872953 | 0.28558708 |
| ANG | -1.3476143 | 0.00797106 | 0.16256462 |
| ANKRD2 | 1.42029258 | 0.04158174 | 0.33631967 |
| SIGLEC1 | 1.63418153 | 0.00412912 | 0.11556518 |
| HPS1 | -3.2759629 | 0.00394749 | 0.11434218 |
| ATP13A1 | 3.3358189 | 7.32E-05 | 0.02889205 |
| CLEC3B | -1.1800391 | 0.01759834 | 0.23307063 |
| PTK7 | 1.66440651 | 0.04752542 | 0.35777959 |
| GSTT1 | -2.0639066 | 0.0368671 | 0.31624683 |
| CCL16 | -2.4111808 | 0.00170414 | 0.08761099 |
| PXDN | -1.6368923 | 0.01174435 | 0.19179501 |
| P4HA1 | 3.86393803 | 0.00236074 | 0.09253963 |
| HTRA1 | -1.0197832 | 0.00288766 | 0.09955536 |
| PMPCA | 1.32619768 | 0.01304432 | 0.2053062 |
| GSTM2 | 2.39619794 | 0.01275576 | 0.20264939 |
| IGF2 | -1.0152445 | 0.00878565 | 0.16594387 |
| CAPNS1 | 1.03727674 | 0.0083994 | 0.16391523 |
| UPF1 | 1.24175247 | 0.03331106 | 0.30463841 |
| APOL2 | 1.87825464 | 0.01009043 | 0.17746077 |
|  | 1.71743877 | 0.04492668 | 0.35037968 |
| TUBAL3 | 1.96201337 | 0.0351304 | 0.3101757 |
| RPAP3 | 2.1637308 | 0.02692326 | 0.28456312 |
| DENR | -1.6274836 | 3.62E-05 | 0.02621076 |
| CST3 | -1.1314997 | 0.03200894 | 0.30264907 |
| COLEC10 | -1.1177045 | 0.02166191 | 0.25570472 |
| SGTA | 1.17208399 | 0.03260214 | 0.30395566 |
| SRRM2 | 2.70250641 | 0.00068402 | 0.06085176 |
| PAK1 | 2.07937558 | 0.00137643 | 0.08153053 |
| IGFBP7 | -1.3784608 | 0.00653402 | 0.14783227 |
| RANBP1 | -1.0024818 | 0.00615964 | 0.14530851 |
| SDCBP | -2.4837104 | 0.00132269 | 0.0799415 |
| ECRG4 | -1.7871339 | 0.03954059 | 0.32668303 |
| CIDEB | -1.2802009 | 0.03182343 | 0.30249663 |
| GYPC | -2.929248 | 0.03796818 | 0.32088284 |
| FLNC | 2.44414794 | 0.04212822 | 0.33764761 |
| HSP90AB2P | 1.05148058 | 0.0009541 | 0.06794423 |
| LOXL1 | -1.6319916 | 0.00188067 | 0.08761099 |
| MCCC2 | 1.31659065 | 0.0468206 | 0.35682224 |
| IGFBP5 | -1.4033571 | 0.00553818 | 0.13591997 |
| CARHSP1 | -1.4173358 | 0.01487634 | 0.21287511 |
| CAPRIN1 | 1.2832396 | 0.01329207 | 0.20695611 |
| RAD23B | 2.14487331 | 0.04580249 | 0.35466312 |
| COL1A2 | -1.8950566 | 0.02010548 | 0.24852103 |
| PRSS50 | 1.8958534 | 0.00447332 | 0.11921544 |
| GSS | 1.6559816 | 0.00714601 | 0.15676278 |
| ACTA1 | 1.94457871 | 0.01975688 | 0.24622634 |
| SRSF1 | 1.11411231 | 0.01165813 | 0.19110527 |
| PDXP | -2.2866907 | 0.0021958 | 0.08993516 |
| GNPTG | -1.2122592 | 0.04873672 | 0.35777959 |
| SSB | -1.6517697 | 0.02141131 | 0.25482397 |
| EHHADH | 2.0609867 | 0.0360621 | 0.3145658 |
| CHGA | -2.0740947 | 0.03938099 | 0.32668303 |
| MAP1A | 3.89036715 | 0.0073551 | 0.15749312 |
| CCL18 | -1.1270366 | 0.0050312 | 0.12817854 |
| CFHR3 | -1.5561721 | 0.03699452 | 0.31634681 |
| HDGFL2 | 2.72395419 | 0.0003347 | 0.05013649 |
| TBL2 | 3.69184832 | 0.00147599 | 0.08326867 |
| DLGAP4 | 2.4992536 | 0.02805548 | 0.28558708 |
| SORL1 | -1.3829453 | 0.04145807 | 0.33631967 |
| MFAP5 | -2.0807785 | 0.01200709 | 0.19318075 |
| PRAF2 | 1.48710208 | 0.02370721 | 0.26830171 |
| SVEP1 | 1.01074106 | 0.03677473 | 0.31624683 |
| FKBP15 | 1.19758215 | 0.04246022 | 0.33895993 |
| PCDH1 | -1.5738846 | 0.02514856 | 0.27797803 |
| SPG11 | 2.17558844 | 0.02801458 | 0.28558708 |
| ATG4B | 1.57720766 | 0.00581565 | 0.1403509 |
| KCNK6 | 1.93371018 | 0.03549801 | 0.31215252 |
| GPLD1 | -2.063445 | 0.00175092 | 0.08761099 |
| ZC3H18 | 1.58127637 | 0.005123 | 0.12863761 |
| GZMK | -4.7913462 | 0.00019467 | 0.03914475 |
| PPM1B | 3.06964245 | 0.00064886 | 0.06085176 |
| MTOR | 1.32107908 | 0.03409917 | 0.30604707 |
| HAGH | -1.5454545 | 0.00057758 | 0.06085176 |
| ARFGEF1 | 1.47038851 | 0.00727894 | 0.15731209 |
| IER5 | -1.1023209 | 0.02371728 | 0.26830171 |
| SMCR8 | -2.7025338 | 0.00267856 | 0.09616237 |
| REEP4 | 1.86566848 | 0.00184022 | 0.08761099 |
| EPX | -4.3827174 | 0.00023529 | 0.03931168 |
| CTSS | -1.690329 | 0.00015283 | 0.03914475 |
| CMPK2 | 2.57022865 | 0.00176496 | 0.08761099 |
| PCOLCE2 | -1.8643596 | 0.00066303 | 0.06085176 |
| SLC2A1 | -2.2750566 | 0.04245692 | 0.33895993 |
| UBAC2 | 1.86525812 | 0.01311816 | 0.20572309 |
| RAB11FIP3 | 1.06927183 | 0.0402964 | 0.3315295 |
| FAH | 2.74008113 | 0.0381719 | 0.32155473 |
| ADSL | -1.3930678 | 0.00094376 | 0.06794423 |
| GLRX5 | 2.36312472 | 0.04116187 | 0.33485469 |
| COLEC11 | -1.1272862 | 0.02131891 | 0.25442126 |
| REG4 | -2.0986662 | 0.00258014 | 0.09435686 |
| SERPINF1 | -1.0324079 | 0.0043572 | 0.11756314 |
| INPP4B | 1.441651 | 0.02872964 | 0.28558708 |
| PROS1 | -1.3751829 | 0.001325 | 0.0799415 |
| CA1 | -1.6526588 | 0.02033466 | 0.24856066 |
| PRKAR2A | 1.46190269 | 0.0417958 | 0.33684777 |
| GPR108 | 1.27767731 | 0.02946904 | 0.28962337 |
| GLTPD2 | -1.1672782 | 0.01351607 | 0.2074693 |
| SRSF5 | 1.52807433 | 0.00313962 | 0.10102587 |
| NUCB1 | 1.40194289 | 0.01212234 | 0.19431534 |
| EPPK1 | 2.00114455 | 0.04342847 | 0.34238343 |
| DNAJA4 | -1.5440687 | 0.01000953 | 0.17675362 |
| FAT1 | -1.7979305 | 0.04423324 | 0.34621479 |
| AUP1 | 1.08604584 | 0.02838739 | 0.28558708 |
| COMMD3 | 1.42514126 | 0.0164841 | 0.22447308 |
| PYCR1 | 2.00427422 | 0.04884054 | 0.35777959 |
| GMIP | 1.45330043 | 0.02813022 | 0.28558708 |
| TXNDC12 | 1.63602525 | 0.0334795 | 0.30505732 |
| IGF2BP3 | 1.04461946 | 0.0473942 | 0.35777959 |
| KLHDC4 | 2.43956667 | 0.00180533 | 0.08761099 |
| LUC7L2 | 1.02079459 | 0.03271268 | 0.304291 |
| CXCL2 | 2.41396788 | 6.65E-05 | 0.02889205 |
| TAGLN | -1.0108601 | 0.01143944 | 0.18966758 |
| DDX58 | 2.92385435 | 0.00198751 | 0.0880995 |
| KEL | -3.4668315 | 0.036679 | 0.31624683 |
| GALNT16 | -2.2593217 | 0.00529381 | 0.13216281 |
| VCL | 1.78558521 | 0.03219851 | 0.30264907 |
| ACKR1 | -1.8325273 | 0.00016998 | 0.03914475 |
| CHST7 | -1.9240749 | 0.02058589 | 0.24951803 |
| TCEA1 | -1.4551387 | 0.04210859 | 0.33764761 |
| VRK1 | 1.12048179 | 0.02594332 | 0.28316022 |
| HSPH1 | 2.04859517 | 0.04467325 | 0.34902988 |
| NFKB1 | 1.76308103 | 0.00735983 | 0.15749312 |
| EHD1 | -1.4870926 | 0.00477711 | 0.12501053 |
| MTCH1 | 1.30611293 | 0.01643715 | 0.22447308 |
| ANGPTL6 | -1.0814063 | 0.04165285 | 0.33631967 |
| LNPEP | -1.5084266 | 0.03954693 | 0.32668303 |
| PI4K2A | -2.2255316 | 0.03212976 | 0.30264907 |
| FAF1 | 2.14928732 | 0.00226689 | 0.09117949 |
| ECI1 | 1.61450329 | 0.04815105 | 0.35777959 |
| ARL6IP4 | 1.34182927 | 0.02013798 | 0.24852103 |
| CRTAC1 | -1.1749828 | 0.0033588 | 0.10352444 |
| ARPC5L | 1.4761595 | 0.02707427 | 0.28546272 |
| ETF1 | 1.59245855 | 0.03107481 | 0.29864817 |
| PSMC4 | 1.40055437 | 0.01579768 | 0.22129312 |
| U2SURP | -2.8823596 | 0.01200629 | 0.19318075 |
| CNRIP1 | -1.8289994 | 0.00499695 | 0.12817854 |
| SESTD1 | 1.56160017 | 0.01130153 | 0.18882242 |
| CXCL13 | 2.02289689 | 0.01949488 | 0.24593232 |
| BAX | 2.34781935 | 0.00070041 | 0.06085176 |
| MRE11 | -1.8962278 | 0.01417088 | 0.20898731 |
| AHSP | -3.1062565 | 0.0003337 | 0.05013649 |
| ARPC1A | 2.04950034 | 0.00204677 | 0.08891172 |
| CDH6 | -1.8056927 | 0.00256987 | 0.09435686 |
| FBXO7 | -1.4709023 | 0.01361339 | 0.20822741 |
| NARS1 | -1.8484853 | 0.00293095 | 0.1001528 |
| LEP | -3.4521144 | 0.00841462 | 0.16391523 |
| PBXIP1 | -1.7646309 | 0.00108998 | 0.07205381 |
| BPIFB1 | -1.7042876 | 0.00422257 | 0.11592883 |
| PPIL4 | -1.8551814 | 0.01114684 | 0.18841188 |
| CHST14 | -1.4930527 | 0.0091038 | 0.16616352 |
| MAT2B | 2.08301707 | 0.00890076 | 0.16594387 |
| VIT | -2.1326821 | 0.00341714 | 0.10453553 |
| DKC1 | 2.46681821 | 0.02511879 | 0.27797803 |
| KLK14 | -1.2684179 | 0.00101727 | 0.07014347 |
| COPB1 | 1.79005603 | 0.03388106 | 0.30598609 |
| GAL | -1.8553917 | 0.00019825 | 0.03914475 |
| DENND4C | 2.52039713 | 0.00628335 | 0.14530851 |
| HRG | -1.4275829 | 0.00045849 | 0.05651512 |
| FTCD | -1.6678141 | 0.02652114 | 0.28455106 |
| FABP1 | -1.6413257 | 0.01021708 | 0.17824491 |
| STAB2 | -2.2610756 | 0.00705539 | 0.15637045 |
| MAN1A2 | -1.2790807 | 0.01549419 | 0.21852838 |
| NDUFC1 | 1.34701158 | 0.03062669 | 0.29662074 |
| AK1 | -1.1895503 | 0.02656672 | 0.28455106 |
| PRPS1 | -2.2576162 | 5.09E-05 | 0.02889205 |
| SERPINE1 | -2.6985528 | 7.27E-05 | 0.02889205 |
| PDXDC1 | 1.73022159 | 0.01108103 | 0.18803129 |
| MYL4 | -1.5661286 | 0.01400702 | 0.20898731 |
| ACOT7 | 1.13991357 | 0.04907208 | 0.35887055 |
| TSC22D1 | 1.24198192 | 0.01456768 | 0.21234112 |
| FRG1 | 2.55526262 | 0.00789724 | 0.16181884 |
| EIF5A | -1.4849653 | 0.02457043 | 0.2750875 |
| IFI6 | 2.09932517 | 0.00336025 | 0.10352444 |
| SNX9 | 1.92772499 | 0.04868147 | 0.35777959 |
| COL15A1 | -2.6050143 | 0.00077417 | 0.06319625 |
| DNAJB4 | -2.0337352 | 0.02029516 | 0.24856066 |
| SEMA3B | -2.0840804 | 0.00058609 | 0.06085176 |
| MSLN | -1.3799453 | 0.00404929 | 0.11556518 |
| PPP2CB | 1.39548276 | 0.00876004 | 0.16594387 |
| MYOT | 1.45077532 | 0.04393271 | 0.34475135 |
| INHBE | -1.4263862 | 0.00047951 | 0.05651512 |
| OLFML3 | -1.4902538 | 0.00753552 | 0.15868707 |
| PEBP4 | -3.0449043 | 1.70E-05 | 0.0246801 |
| PRKAA1 | 2.08827437 | 0.03240576 | 0.30338499 |
| ANGPTL1 | -1.4461724 | 0.0362058 | 0.31518637 |
| CXCL10 | 3.73282388 | 2.31E-05 | 0.02504269 |
| MMRN2 | -1.1090397 | 0.04833272 | 0.35777959 |
| CCN5 | -2.3682834 | 0.0001855 | 0.03914475 |
| SARS2 | 1.44631025 | 0.01584304 | 0.22129312 |
| PNPO | 3.4299915 | 3.16E-05 | 0.02621076 |
| CBX1 | 1.2597664 | 0.00887454 | 0.16594387 |
| LRPPRC | 3.0475542 | 0.00076186 | 0.06319625 |
| ZW10 | 2.22043958 | 0.03260666 | 0.30395566 |
| METRNL | -1.9706949 | 0.00189211 | 0.08761099 |
| F8 | -1.2318233 | 0.00150959 | 0.08407237 |
| TAC3 | -1.1349193 | 0.02866932 | 0.28558708 |
| WDR44 | 1.30300201 | 0.04598344 | 0.3547994 |
| OXSR1 | -1.4240465 | 0.00040933 | 0.0550715 |
| PML | 2.59029145 | 0.03963213 | 0.32668303 |
| PYGL | 3.62742847 | 0.00286799 | 0.09955536 |
| PCDH7 | -2.2018799 | 0.01372166 | 0.20862349 |
| NMI | 2.20097318 | 0.00174883 | 0.08761099 |
| TLL2 | -1.3629017 | 0.00155328 | 0.08541065 |
| DMKN | -1.8763054 | 0.01498717 | 0.21287511 |
| TIMM44 | 1.25656054 | 0.020822 | 0.25091566 |
| AGTRAP | 4.28411159 | 0.03728735 | 0.31714231 |
| VWA5A | 3.14026202 | 0.03166908 | 0.30168964 |
| FCN3 | 1.07930569 | 0.04563328 | 0.3539839 |
| INHBC | -1.0930663 | 0.0013988 | 0.08153053 |
| TNFRSF17 | -2.2865555 | 0.00500234 | 0.12817854 |
| ANKS1A | 1.82673314 | 0.03150094 | 0.30102859 |
| TRDN | -1.2346403 | 0.01379766 | 0.20862349 |
| PRPF4B | 2.76340495 | 0.00030342 | 0.04881676 |
| MTHFR | 2.12472914 | 0.00839919 | 0.16391523 |
| VBP1 | 1.60869628 | 0.00321595 | 0.10186701 |
| MICU2 | 1.6422327 | 0.02691412 | 0.28456312 |
| PPCS | 3.316205 | 0.00061563 | 0.06085176 |
| XRN1 | 1.27456382 | 0.00323013 | 0.10186701 |
| TRIP10 | 1.63158159 | 0.03396237 | 0.30604707 |
| RIC8A | -1.490083 | 0.00190518 | 0.08761099 |
| F5 | -1.0292936 | 0.0082505 | 0.16391523 |
| GPCPD1 | 1.20207683 | 0.01413599 | 0.20898731 |
| API5 | 2.65234871 | 0.02351015 | 0.26805275 |
| GBF1 | 1.59265917 | 0.01890776 | 0.24204968 |
| PRRC1 | 1.62532882 | 0.04996884 | 0.36359237 |
| DRAP1 | 1.11614137 | 0.02200043 | 0.2582969 |
| PRPSAP1 | -1.7186651 | 0.00041836 | 0.0550715 |
| THRAP3 | 1.79718991 | 0.00712364 | 0.15676278 |
| MMGT1 | 1.0202092 | 0.01265008 | 0.20202927 |
| HLA-DRB1 | 1.13934019 | 0.02085188 | 0.25091566 |
| C1QTNF3 | -1.749593 | 0.00630899 | 0.14530851 |
| PSMB10 | 1.34291158 | 0.0024093 | 0.09261961 |
| SOST | -1.4790291 | 0.00372035 | 0.11069319 |
| CXCL11 | 3.64682992 | 1.02E-05 | 0.02204773 |
| CDH13 | -1.9193954 | 0.02811975 | 0.28558708 |
| AEBP1 | -1.2020454 | 0.04639703 | 0.35546504 |
| MIA3 | 1.75814295 | 0.04275082 | 0.33979715 |
| CNOT1 | 2.01108714 | 0.00019157 | 0.03914475 |
| MGAT5 | -2.1613419 | 0.0007803 | 0.06319625 |
| TTPA | -1.5717896 | 0.01461556 | 0.21234112 |
| ADH1A | -1.672198 | 0.03864009 | 0.32279336 |
| PHPT1 | 2.16536535 | 0.0068561 | 0.15273275 |
| CRIP1 | -1.0824517 | 0.00300374 | 0.10037127 |
| CCM2 | 1.73482665 | 0.04822108 | 0.35777959 |
| MPC2 | 1.95270414 | 0.04111071 | 0.33485469 |
| HEBP1 | -1.217787 | 0.03015761 | 0.29439251 |
| ALDOA | 1.389567 | 0.03830964 | 0.32188991 |
| IFI27 | 2.89553057 | 0.00168696 | 0.08761099 |
| HNRNPA3 | -1.6416264 | 0.02625041 | 0.28443498 |
| NCKAP1 | 1.91045587 | 0.00632212 | 0.14530851 |
| KIF15 | 2.68750443 | 0.00274854 | 0.09786608 |
| ANGPTL4 | -1.4290325 | 0.02923731 | 0.28837154 |
| DNPEP | 2.9184321 | 0.00037805 | 0.05474198 |
| PRDX2 | -1.3721955 | 0.00212377 | 0.08923727 |
| DNM1 | 1.49558197 | 0.03313984 | 0.30439955 |
| PIK3R1 | 2.50124417 | 0.00264108 | 0.09560695 |
| CCL24 | -1.3735907 | 0.01383139 | 0.20862349 |
| VGF | -1.5820572 | 0.00323611 | 0.10186701 |
| LECT2 | -1.4220945 | 0.00285639 | 0.09955536 |
| PRG2 | -2.8510062 | 0.01415068 | 0.20898731 |
| GBP2 | 1.71068473 | 0.00140764 | 0.08153053 |
| F8A3 | 2.06269 | 0.00067179 | 0.06085176 |
| GAL3ST4 | -1.9399042 | 0.00018544 | 0.03914475 |
| NUDT16 | -1.6518274 | 0.00580228 | 0.1403509 |
| CHKB | 1.36645083 | 0.00759828 | 0.15868707 |
| HCLS1 | 2.15229566 | 0.00220453 | 0.08993516 |
| WARS1 | 2.03712024 | 0.00118493 | 0.07682586 |
| LIPC | -1.9056119 | 0.02114736 | 0.25306929 |
| EML2 | 2.10204356 | 0.0219622 | 0.2582969 |
| RANBP9 | 1.18269784 | 0.02966454 | 0.2908866 |
| SPTLC2 | 1.13602207 | 0.01636985 | 0.22447308 |
| ADD1 | -1.0308843 | 0.01843646 | 0.23906861 |
| GNLY | -1.0445684 | 0.02157652 | 0.25539072 |
| BANK1 | 2.0584004 | 0.02795818 | 0.28558708 |
| UBA2 | 1.52265378 | 0.04206157 | 0.33764761 |
| PAIP1 | 2.81946892 | 0.01408759 | 0.20898731 |
| NPEPL1 | 1.39291983 | 0.04512496 | 0.35129538 |
| TBC1D13 | 1.71117211 | 0.01350764 | 0.2074693 |
| SRRM1 | 1.7264055 | 0.04840488 | 0.35777959 |
| TIMP2 | -1.3217651 | 0.03688774 | 0.31624683 |
| CYTL1 | -1.9554757 | 0.01702662 | 0.22832196 |
| ABRACL | 1.87298762 | 0.00282496 | 0.09955536 |
| SELENOP | -1.0854939 | 0.00221525 | 0.08993516 |
| SCGB1A1 | -1.5668495 | 0.04734254 | 0.35777959 |
| FRMD8 | 1.02774708 | 0.02037007 | 0.24856066 |
| EPB41 | -1.0634625 | 0.03442301 | 0.30700994 |
| FUNDC1 | 1.52683005 | 0.00589005 | 0.14136112 |
| CCL14 | -1.5479918 | 0.01944302 | 0.24593232 |
| RBM3 | -1.2309004 | 0.00209023 | 0.08923727 |
| TNNT2 | 2.5808571 | 0.01101923 | 0.18771575 |
| PTN | 1.2056941 | 0.03573395 | 0.31233052 |
| CILP | 1.74027921 | 0.03691002 | 0.31624683 |
| CFHR2 | -1.5193807 | 0.01608975 | 0.22330317 |
| TEX2 | 1.41247835 | 0.00964024 | 0.17233415 |
| EIF4B | -1.2409475 | 0.00018417 | 0.03914475 |
| TCOF1 | 2.66283053 | 0.00063506 | 0.06085176 |
| CCDC126 | -1.0598612 | 0.01080346 | 0.18623115 |
| OLFML1 | -1.8730219 | 0.01059968 | 0.18344623 |
| NUMB | 1.72361896 | 0.00635902 | 0.14538727 |
| PIN1 | -1.2929608 | 0.00021842 | 0.03931168 |
| OAS2 | 1.30595855 | 0.00878092 | 0.16594387 |
| SNX18 | 1.86647907 | 0.00190514 | 0.08761099 |
| YBX3 | -1.4786847 | 0.0096845 | 0.17241593 |
| CEMIP | -2.1751903 | 0.00185152 | 0.08761099 |
| TFPI | -1.3940829 | 0.00426994 | 0.11592883 |
| NFU1 | 1.85641207 | 0.00090755 | 0.06794423 |
| SRSF4 | 1.22729167 | 0.00415013 | 0.11556518 |
| TUBB6 | 1.51775366 | 0.04396691 | 0.34475135 |
| ADAM15 | -2.1390474 | 0.00203483 | 0.08891172 |
| WASHC5 | 1.41146883 | 0.02522301 | 0.27809332 |
| DLG1 | 1.78587597 | 0.01629732 | 0.22447308 |
| IRF9 | 1.86842448 | 0.02692344 | 0.28456312 |
| ABHD14B | 1.39403169 | 0.02235976 | 0.26110434 |
| PPP1R21 | 2.7618653 | 0.03365213 | 0.30505732 |
| ADH4 | -2.4600353 | 0.00194366 | 0.08761099 |
| COX20 | -1.5269305 | 0.02005601 | 0.24852103 |
| PRG4 | -1.3498503 | 0.00061143 | 0.06085176 |
| LSM 4.00 | 2.4716401 | 0.0001138 | 0.03802558 |
| SIL1 | -1.5024139 | 0.00986903 | 0.17498393 |
| FUBP1 | 1.05285793 | 0.00776101 | 0.16054199 |
| DSC3 | -2.2451923 | 0.0061028 | 0.14530851 |
| ACAD8 | 1.25040918 | 0.02362471 | 0.26830171 |
| TPM2 | 3.08998505 | 0.03314479 | 0.30439955 |
| ARL 2.00 | 1.18709598 | 0.00245568 | 0.09357415 |
| HSPB7 | 1.51293217 | 0.03884228 | 0.32385962 |
| DNAJB1 | -1.2552888 | 0.00503211 | 0.12817854 |
| GNL1 | -1.4142502 | 0.02294251 | 0.26411989 |
| NNMT | 1.57569102 | 0.04680855 | 0.35682224 |
| ADD2 | -2.2860489 | 0.00808868 | 0.16342891 |
| HAMP | -2.7147059 | 0.00348684 | 0.10592183 |
| DDX46 | -1.2524541 | 0.00308333 | 0.10070652 |
| MX1 | 2.94258646 | 0.00048137 | 0.05651512 |
| FAM126A | 1.38288408 | 0.03819573 | 0.32155473 |
| BLVRB | -1.4011608 | 0.00237651 | 0.09253963 |
| DEFB1 | -1.8032238 | 0.01925674 | 0.24459432 |
| CIAPIN1 | 2.04766698 | 0.00312888 | 0.10102587 |
| IGHE | 1.05174914 | 0.03681311 | 0.31624683 |
| USP4 | 1.17737689 | 0.02300347 | 0.26411989 |
| C19orf12 | 1.26583261 | 0.01335588 | 0.2072069 |
| SAE1 | -1.4204172 | 0.00299336 | 0.10037127 |
| IFIT1 | 1.21188469 | 0.03409579 | 0.30604707 |
| MFSD10 | 1.00549745 | 0.00834878 | 0.16391523 |
| CAMK2D | 2.33469573 | 0.01859965 | 0.24017995 |
| RARRES2 | -1.532324 | 0.00472419 | 0.12469159 |
| SEC14L5 | 1.78836713 | 0.0082911 | 0.16391523 |
| ATXN10 | 1.64672207 | 0.00258482 | 0.09435686 |
| CRIP2 | -1.5353623 | 0.04323953 | 0.34213575 |
| CDH2 | -1.232812 | 0.00785153 | 0.16164479 |
| HLA-C | 1.49027493 | 0.03686781 | 0.31624683 |
| ISG20 | 1.43176729 | 0.00871459 | 0.16594387 |
| PROC | -1.0179318 | 0.01098024 | 0.18771575 |
| CD59 | -1.2995618 | 0.01013996 | 0.17761291 |
| F11 | -1.551758 | 0.0273793 | 0.28558708 |
| UGP2 | 1.73459532 | 0.00473621 | 0.12469159 |
| TUBB8 | 1.39256165 | 0.00645422 | 0.14679127 |
| DDX60 | 2.56793999 | 0.00090452 | 0.06794423 |
| HMGB1 | 1.37175918 | 0.03730656 | 0.31714231 |
| APOL3 | 1.56987613 | 0.0328129 | 0.30439955 |
| CNDP1 | -1.2373953 | 0.01632115 | 0.22447308 |
| TGFBI | -1.0367085 | 0.00510311 | 0.12863761 |
| CHORDC1 | 1.93964153 | 0.01499535 | 0.21287511 |
| GCDH | 1.5702514 | 0.03724258 | 0.31714231 |
| PTGES2 | 1.45422169 | 0.02304361 | 0.26411989 |
| LANCL2 | -1.3645293 | 0.00411754 | 0.11556518 |
| SMAD2 | 1.27500155 | 0.01426653 | 0.20937095 |
| MGP | -1.8046241 | 0.00039835 | 0.0550715 |
| DDI2 | -1.6497336 | 0.03314002 | 0.30439955 |
| ARR3 | 1.01722232 | 0.04733322 | 0.35777959 |
| PDHX | 1.50231656 | 0.00365689 | 0.1097001 |
| COX16 | -1.3588393 | 0.02856599 | 0.28558708 |
| TRIM21 | 1.87778779 | 0.03959124 | 0.32668303 |
| AIDA | -1.2814539 | 0.00195632 | 0.08761099 |
| SNRPB2 | 1.05765661 | 0.0196325 | 0.2461888 |
| SNX17 | 1.06576903 | 0.00869501 | 0.16594387 |
| SLC25A12 | 1.55351372 | 0.04876317 | 0.35777959 |
| MMP15 | -3.4093049 | 0.00019162 | 0.03914475 |
| EPB42 | -4.2462428 | 0.00195492 | 0.08761099 |
| GCG | -1.9958176 | 0.00426496 | 0.11592883 |
| SERPIND1 | -1.0179638 | 0.00840963 | 0.16391523 |
| GABARAPL2 | -2.1475981 | 0.00560653 | 0.13682463 |
| AKR1A1 | 2.64129032 | 0.04594476 | 0.3547994 |
| FAM180A | -1.6235403 | 0.01685513 | 0.22738722 |
| ERMP1 | 1.19125572 | 0.04761068 | 0.35777959 |
| CASP6 | 2.64963485 | 0.0025034 | 0.0937479 |
| CACNA2D1 | 2.71162973 | 0.02639849 | 0.28455106 |
| NDUFB4 | 1.56777796 | 0.0286498 | 0.28558708 |
| TNXB | -1.5404006 | 0.01139123 | 0.18959194 |
| HMBS | -1.2353966 | 0.03447647 | 0.30700994 |
| ELN | -1.425257 | 0.01492115 | 0.21287511 |
| OSBPL8 | 1.94678887 | 0.02479599 | 0.27689916 |
| NAP1L4 | -1.3148672 | 0.03079557 | 0.29662074 |
| PIK3IP1 | -2.1366501 | 0.00108226 | 0.07205381 |
| NPNT | -1.126033 | 0.01791843 | 0.23445073 |
| SH3BGRL3 | 1.32796163 | 0.03795802 | 0.32088284 |
| NSDHL | 1.97657857 | 0.00445296 | 0.11921544 |
| SHH | 3.2667989 | 0.01089947 | 0.18714344 |
| PTRH2 | 1.92402346 | 0.04164669 | 0.33631967 |
| BIN1 | 3.15250152 | 0.0030777 | 0.10070652 |
| ACHE | -3.0572213 | 0.00023182 | 0.03931168 |

**Table S2. Direct comparison of OAS3 with representative interferon-stimulated proteins in plasma proteomic analysis.**

| Gene symbol | log₂ fold change | P value | P adj |
| --- | --- | --- | --- |
| OAS3 | 3.91 | 6.64e-06 | 0.022 |
| ISG15 | 5.65 | 9.31e-04 | 0.068 |
| MX1 | 2.94 | 4.81e-04 | 0.057 |
| IFIT1 | 1.21 | 0.034 | 0.306 |
| CXCL10 | 3.73 | 2.31e-05 | 0.025 |

Log₂ fold change represents the relative protein abundance in dermatomyositis patients compared with healthy controls.

**Table S3. Individual plasma OAS3 and CK values in patients with dermatomyositis**

| **sample** | **OAS3 (ng/mL)** | **CK (IU/L)** |
| --- | --- | --- |
| **S1** | 2.9925 | 1000 |
| **S2** | 4.5275 | 1569 |
| **S3** | 3.005 | 3699 |
| **S4** | 1.9725 | 651 |
| **S5** | 9.9575 | 3045 |
| **S6** | 0.8475 | 51 |
| **S7** | 10.9775 | 2260 |
| **S8** | 7.4625 | 1260 |
| **S9** | 3.385 | 890 |
| **S10** | 9.4025 | 1670 |
| **S11** | 13.305 | 849 |
| **S12** | 1.41 | 100 |
| **S13** | 5.6875 | 908 |
| **S14** | 8.9 | 3771 |

CK: creatine kinase, OAS3: 2′-5′-oligoadenylate synthetase 3

**Table S4. Multivariable linear regression analysis of plasma OAS3 and serum CK adjusted for an interferon composite score**

| **Variable** | **β coefficient** | **Standard error** | **95% CI** | **P value** |
| --- | --- | --- | --- | --- |
| Intercept | 5.133 | 0.534 | 3.958 to 6.309 | <0.0001 |
| OAS3 | 0.0929 | 0.0722 | −0.0660 to 0.2518 | 0.2248 |
| IFN composite score | 0.000256 | 0.000107 | 2.10×10⁻⁵ to 0.00049 | 0.0354 |

Multivariable linear regression was performed with log-transformed serum creatine kinase as the dependent variable. Independent variables included plasma OAS3 levels and an interferon composite score. The interferon composite score was calculated as the geometric mean of normalized plasma abundances of representative interferon-stimulated proteins (GBP1, EPSTI1, IFI6, CXCL10, IFI27, IFIT5, IFIT1, DDX60, ISG15, SIGLEC1, OAS2, and MX1).

**Model fit:** R² = 0.553, Adjusted R² = 0.471, Overall model P value = 0.012


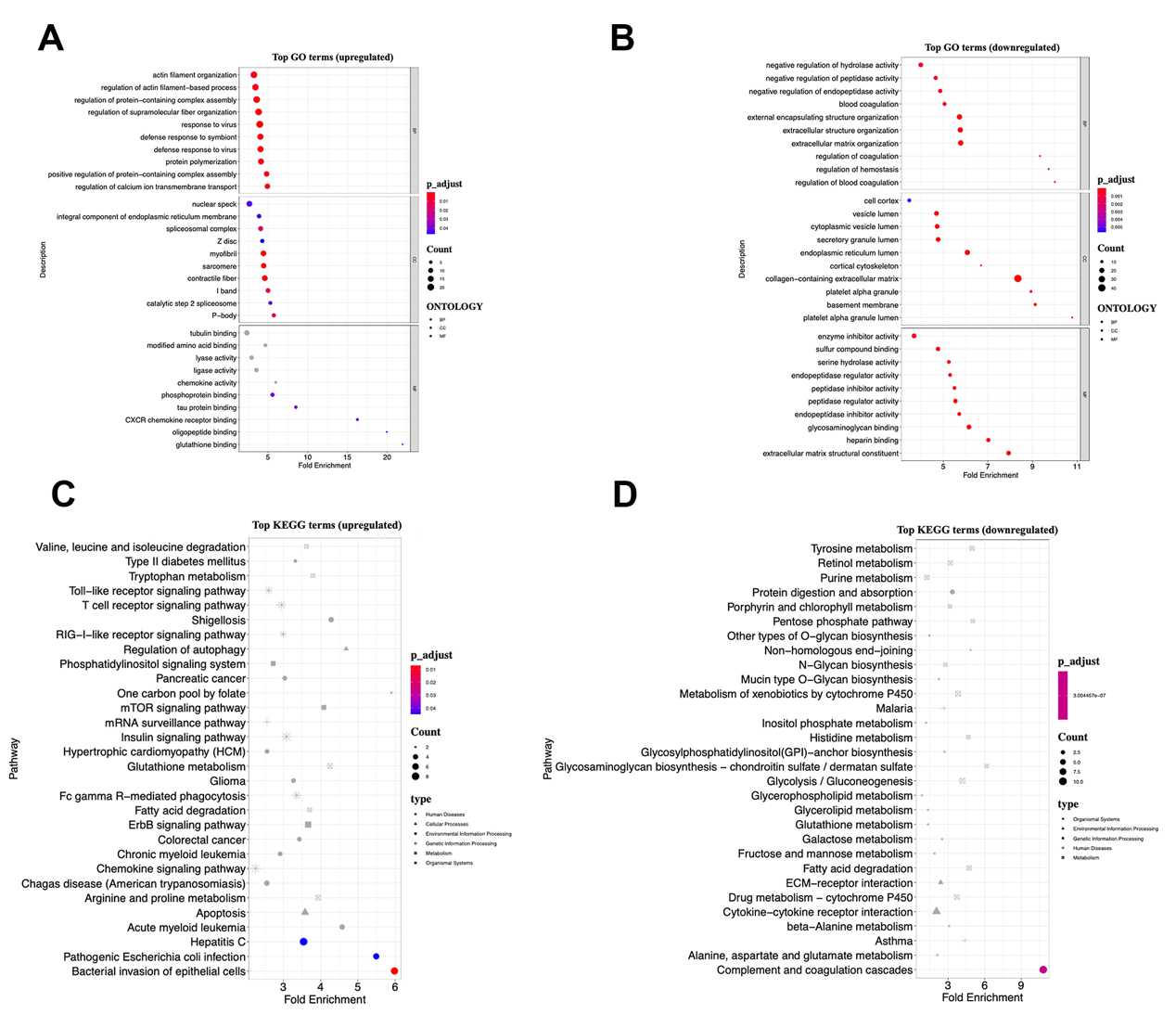


**Figure S1. Full GO and KEGG pathway enrichment analyses of differentially expressed plasma proteins in dermatomyositis.**


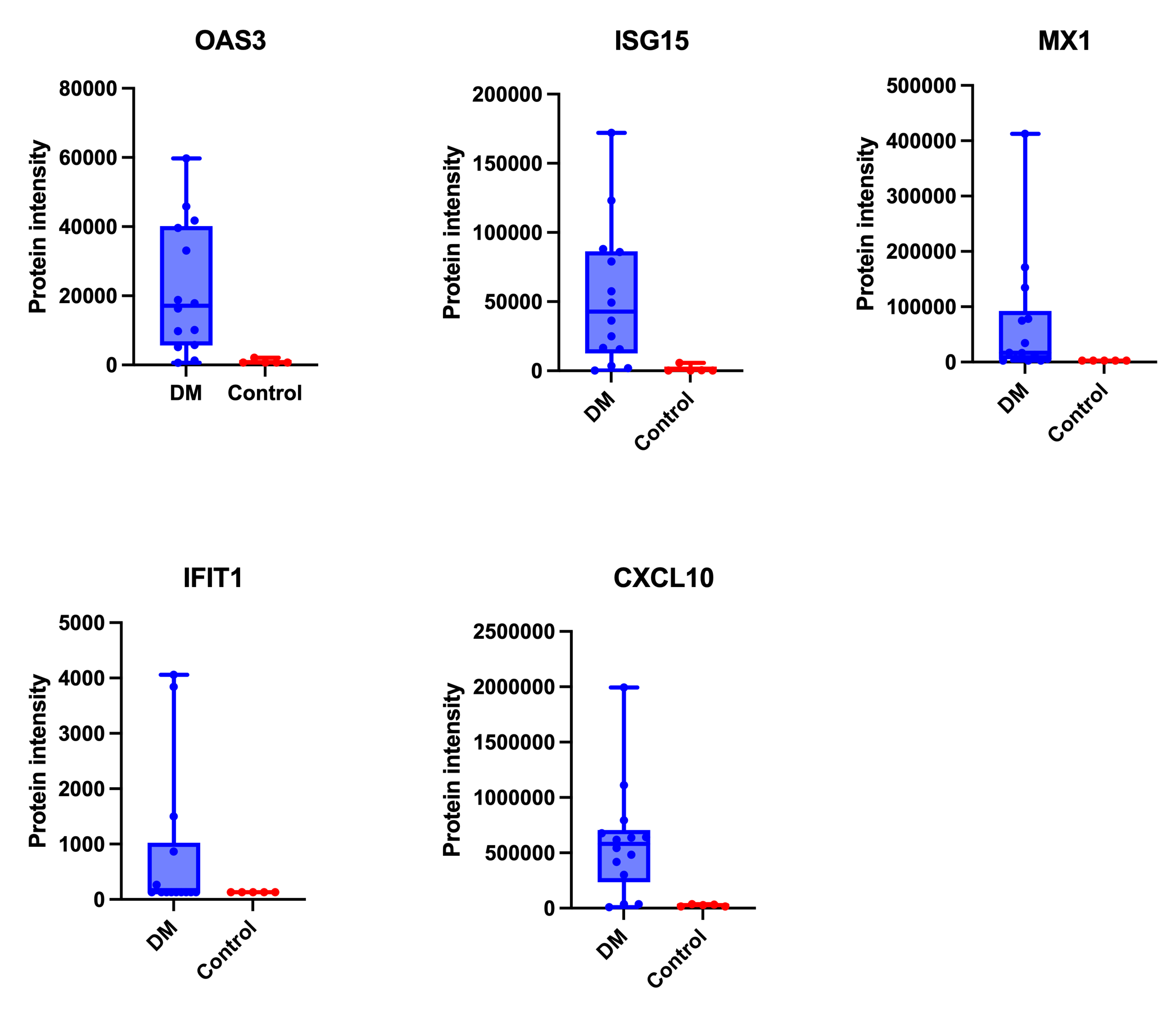


**Figure S2. Plasma protein abundance distributions of OAS3 and representative interferon-stimulated proteins in patients with dermatomyositis and healthy controls.**

Each dot represents an individual sample. Box plots indicate the median and interquartile range.


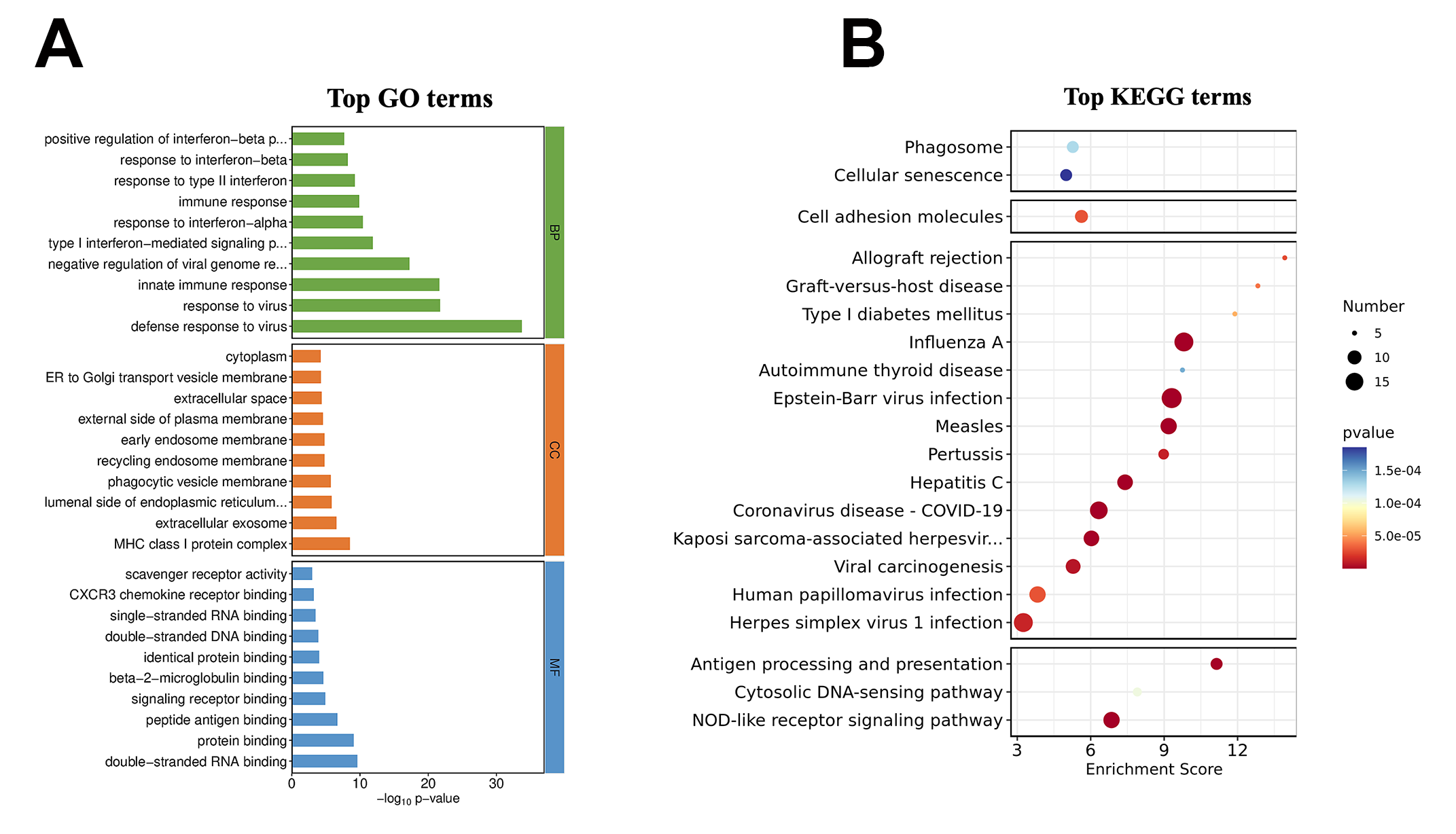


**Figure S3. Full GO and KEGG pathway enrichment analyses of shared upregulated genes in dermatomyositis muscle transcriptomes.**


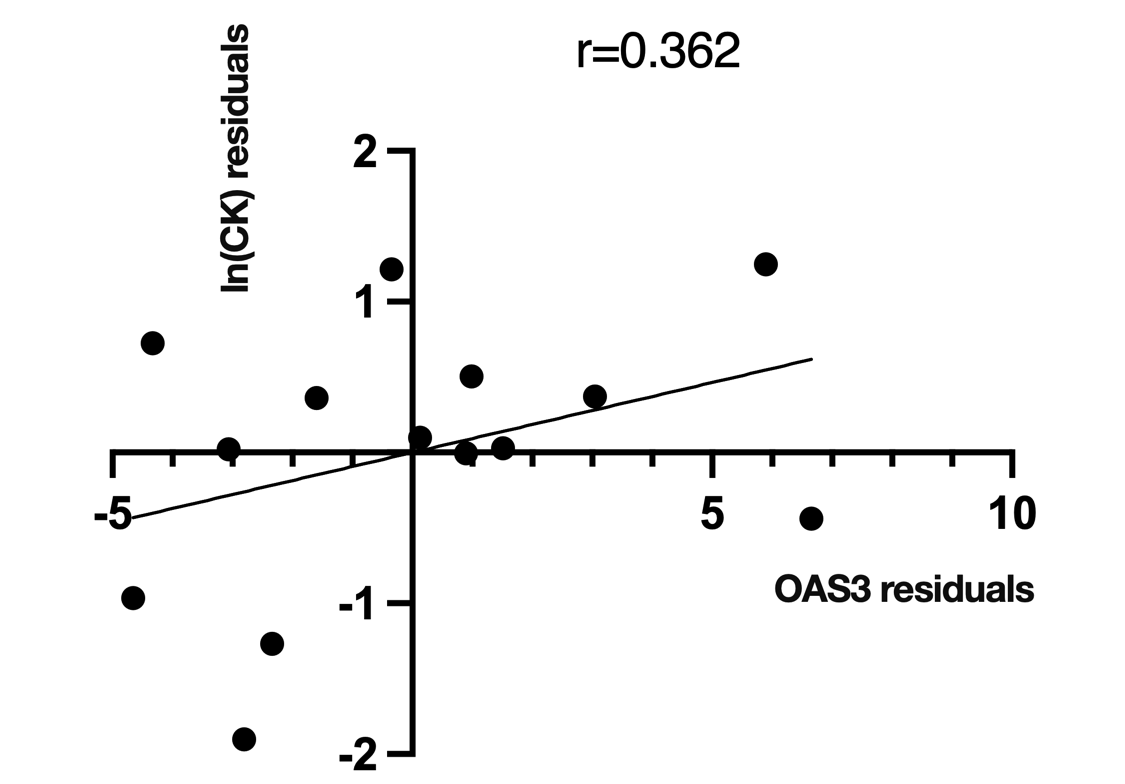


**Figure S4. Partial association between plasma OAS3 levels and serum CK after adjustment for an interferon composite score.**

The interferon composite score was calculated as the geometric mean of normalized plasma abundances of representative interferon-stimulated genes, including GBP1, EPSTI1, IFI6, CXCL10, IFI27, IFIT5, IFIT1, DDX60, ISG15, SIGLEC1, OAS2, and MX1, for each individual sample. Residuals of log-transformed CK and OAS3 were obtained after adjustment for the interferon composite score and used for partial correlation analysis. Each dot represents one patient.
